# A scale assessing doctor-patient communication in a context of acute conditions based on a systematic review

**DOI:** 10.1371/journal.pone.0192306

**Published:** 2018-02-21

**Authors:** Mélanie Sustersic, Aurélie Gauchet, Anaïs Kernou, Charlotte Gibert, Alison Foote, Céline Vermorel, Jean-Luc Bosson

**Affiliations:** 1 Laboratory of « Techniques de l’Ingénierie Médicale et de la Complexité, Informatique, Mathématiques et Applications », University Grenoble Alps, Grenoble, France; 2 Emergency Department, Groupe Hospitalier Mutualiste, Grenoble, France; 3 Laboratory of Psychology, University Grenoble Alps, Grenoble, France; 4 Research Division, Grenoble Alps University Hospital, Grenoble, France; University of Tennessee Health Science Center, UNITED STATES

## Abstract

**Background:**

There is no validated generic tool to measure Doctor-Patient-Communication (DPC) in a context of acute conditions.

**Objective:**

To create and validate such a scale in a real population.

**Materials and method:**

We performed a systematic review of validated DPC scales available in English. From these, using a theoretical model based on a multidisciplinary approach, we selected pertinent items that met the inclusion criteria and included them in a simple questionnaire. This tool based on a synthesis of the literature was then validated in a prospective study in two hospital emergency departments.

**Results:**

We found 22 pertinent questionnaires and scoring systems. From these, we extracted items and built a scale based on 15 questions with graded responses (Likert from 1 to 4). The mean time for questionnaire completion was 3 minutes. We included 189 adults and adolescents in the study and analyzed complete responses to the questionnaire by 149 patients, gathered over the phone one week after their consultation. The scale had high internal consistency (Cronbach’s alpha = 0.89) and good external validity. Two questions were removed due to redundancy giving a scale based on 13 questions.

**Conclusions:**

We have created an easy-to-use and validated generic questionnaire to assess DPC in a context of acute conditions, usable both in clinical research and in routine practice.

## Introduction

Nowadays, effective Doctor Patient Communication (DPC) is considered as an essential part of patient [1] as an important basic skill for doctors [2] that cannot be delegated [3]. The components of DPC have been extensively studied [4–6], especially the relationship between how well the doctor communicates with the patient and the results on the patient’s health [3,7,8]. Good communication has been shown to lead to improvements in symptom relief, clinical outcomes, patient behavior [9–10], and possibly in medication adherence [11]. The assessment of DPC has become a major field of clinical research [12–15].

In a review of the literature about DPC, the three most important aspects were: creating a good interpersonal relationship, exchanging information, and making treatment-related decisions that involve the patients in decision-making [13]. A qualitative study of DPC identified three interacting components (“Listening”, “Asking for information”, and “Giving information”) as central and covering two-thirds of the identified interaction types [16].

Adequate content and an appropriate way of providing information are the founding principles of DPC and contribute to the establishment of a relationship of mutual trust [17].

An acute condition (AC) is often the reason for a primary care consultation (Emergency Department) (ED) or primary care practice) and AC management is becoming a major public health issue [18]. The period after an AC consultation is one of high vulnerability for the patient. The information delivered is crucial [19,20] and could lead to complications if neglected [18] Particularly for AC DPC can be difficult since physicians have restricted time for each patient [20] and don’t already know the patient. The medical communication skills required differ from those required in family practice consultations [18–20]. The expectations of the patient before a ED visit mostly concern 4 themes: understanding the cause and expected trajectory of their symptoms; reassurance; symptom relief; and receiving a plan to manage their symptoms, resolve their issue, or pursue further medical care [21].

Thus, in such a context, Patient Information Leaflets (PILs) given during a consultation could be very helpful in assisting patients make informed choices, take treatments appropriately, or follow advice on lifestyle changes [7,22]. Its communicative effectiveness, considered as the ultimate criterion for the assessment of PILs [22], should be studied more broadly.

In spite of the large number of DPC scales validated over the last forty years and reviews of literature on this topic [13–15], we found no generic scale usable in the context of any acute condition that are well adapted to assess tools such PILs. There are several possible reason for this: either the DPC scale has been developed to assess the competencies of medical students or healthcare providers [4,23,24]; and/or the video or audio recording methods involved are not well suited to an emergency setting where the constraints of time, stress and organization make it necessary to minimize the complexity of the research protocol; and/ or some items were not appropriate in the context of acute conditions (e.g. “Did the doctor ask about all of your health concerns rather than just focusing on the first one you mentioned?" [23]; “The doctor gave me enough chance to talk about all my problems” [25]); and/or some important items were missing (e.g.: "Did the doctor involve you in the decision- making?"[24]), without any scientific justification.

In the medical and psychology literature there is no consensual definition about what DPC is and how it should be measured and no consensus as to a theoretical model that defines the doctor-patient relationship [26], despite the collaboration of experts in the field [5]. Furthermore, definitions of the dimensions to be used (such as ‘trust’, “empathy”) are not unanimous [27], as reflected by the literature search, which uncovered a multitude of heterogeneous scales attempting to characterize DPC.

To fill this gap and to avoid the vagueness of terminology related to this topic, we started from an initial theoretical model we had previously independently developed using a multidisciplinary phenomenological approach [28]. In this model, DPC (e.g. confidence, empathy, reassurance) has psychological, cognitive and emotional effects on patient outcomes (e.g. knowledge, satisfaction, anxiety, behavioral intentions), which impact their behavior (adherence to drug and to non-drug prescriptions, to hygiene and dietary advice; and use of the health care system) and thus on therapeutic outcomes (level of pain, etc.).

Our objective was to develop and validate a DPC scale usable in the context of acute conditions based on a synthesis of the literature and on our theoretical model.

## Materials and methods

### Literature search

**Fig 1:**
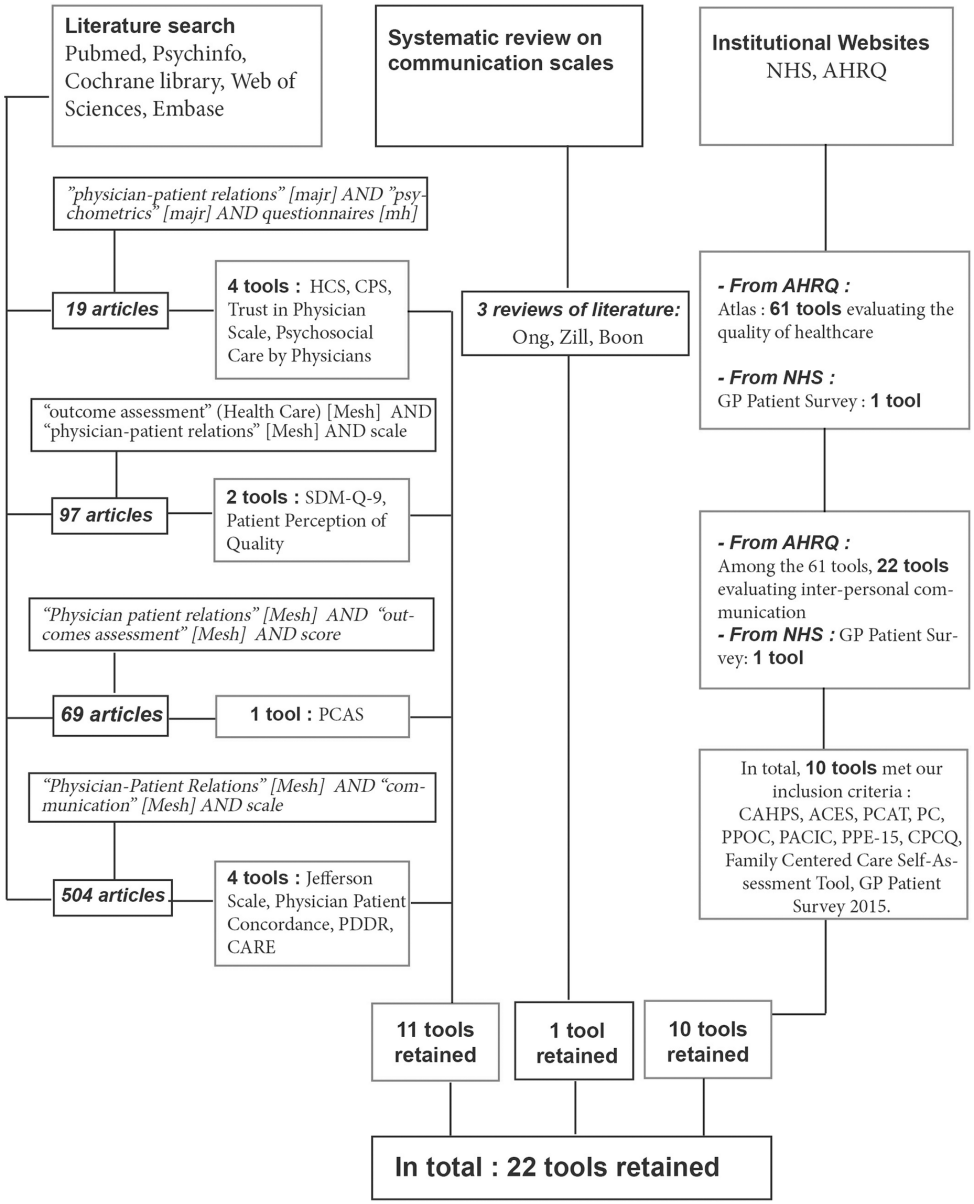
Literature search equations and selection of evaluation tools.

We sought reviews of studies and articles describing validated tools that are currently available to assess DPC during a consultation. Our search covered five databases using four research equations (Fig 1). We restricted articles to those in English published after 1990 for which at least the title and abstract were available. In addition we searched English (NHS) and US (Agency for Healthcare Research and Quality, AHRQ) institutional databases. A manual search was also conducted using the bibliography of selected articles. Searches were last updated in August 2017. Two of the authors independently selected articles, then, together with a third expert, they reassessed the full text of those for which they had not initially concurred.

### Selection criteria for communication scales and items

**Fig 2:**
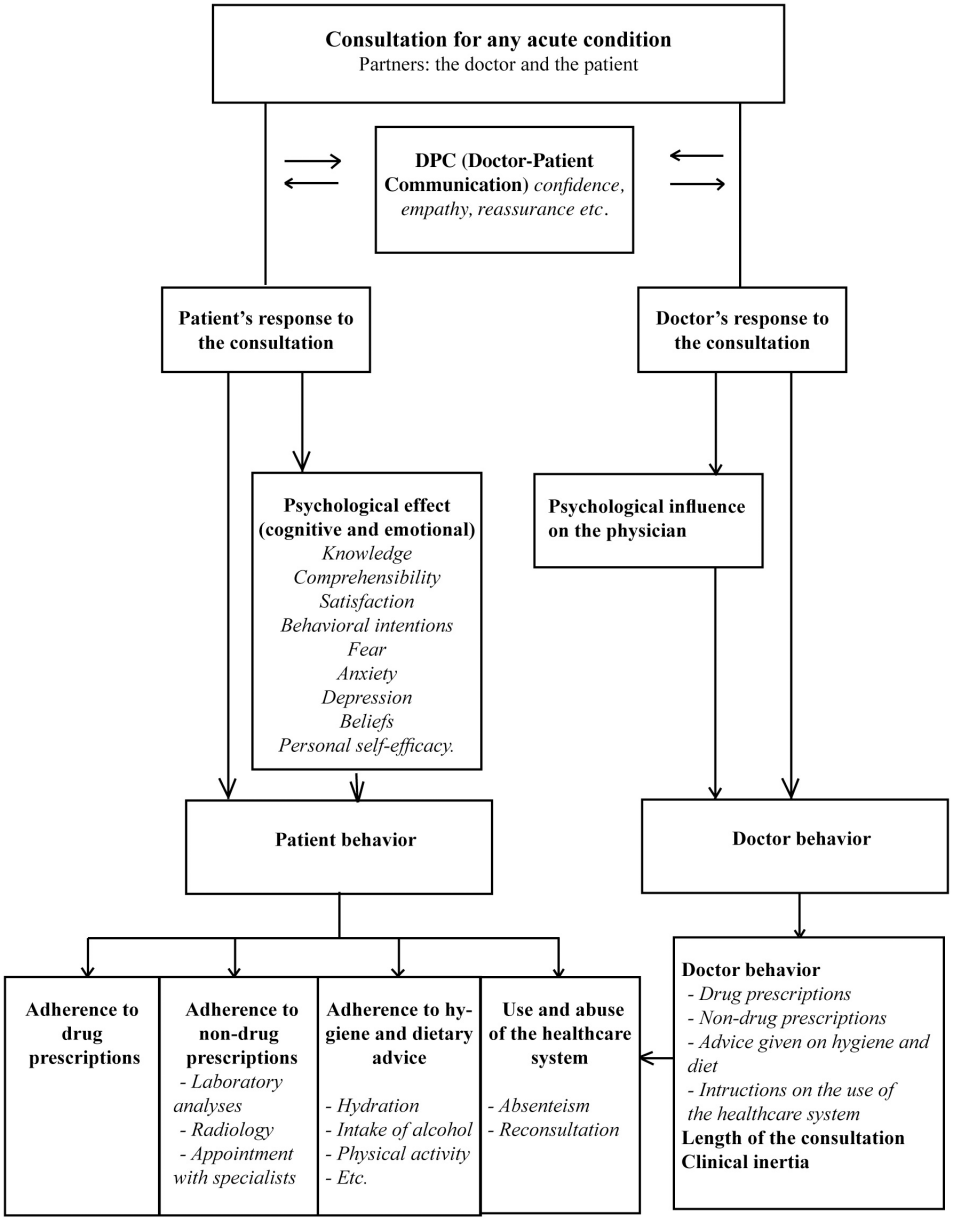
DPC theoretical model adapted to acute conditions.

Starting from the work of Zill et al. and a multidisciplinary model [28] adapted to acute conditions (Fig 2), we drew up a list of inclusion and exclusion criteria see Table 1. The selection criteria focused exclusively on the doctor-patient interaction. Since no single validated scale met all of our criteria, we made a synthesis of items from selected existing scales, avoiding redundancy, to construct a new scale.

**Table 1:** Inclusion and exclusion criteria for scales and items (based on the criteria proposed by Zill et al. and our theoretical model).

**Inclusion criteria for communication scales**
(1) The full text is accessible
(2) The publication is in English
(3) The scale is validated (psychometric properties available)
(4) The scale is self-reported by the patient using a set of closed questions
(5) The measured construct is exclusively communication
(6) The target group is adult patients or adolescents >15 accompanied by an adult
(7) The communication partners are patient and physician
**Exclusion criteria for communication scales**
(1) The scale assesses the communication skills of health professionals
(2) The target group are children or patients’ relatives
(3) Communication is only a subscale of a broader measure
(4) The instrument is condition-specific (i.e.. chronic conditions), specialty-specific, applicable to a subgroup of patients with specific demographic characteristics only (i.e. NOT generic)
(5) The assessment is outside the scope of the consultation (organization of the care system, waiting time etc.)
**Inclusion criteria for items**
(1) Item focuses exclusively on doctor-patient interaction (empathy, decision-making, listening etc.)
(2) Patient centered items
**Exclusion criteria for items**
(1) The item is already included in a scale related to the other outcomes described in the model (satisfaction, patient’s behavior items, adherence etc.).
(2) The Item is intrinsic to the patient (i.e. psychological state of the patient: anxiety, depression, self efficacy etc.)
(3) The Item is intrinsic to the doctor
(4) The item is related to the consultation length
(5) The item requires a more complex protocol than a questionnaire to be measured (i.e. videotape, audiotape)
(6) The measurement of the item poses problems of confidentiality (e.g.video-recording)

### Questionnaire development

The list of selected items was then submitted to a committee of experts made up of doctors, pharmacists, and psychologists (two of whom were bilingual) who pared down the number of items to give a concise, readily useable questionnaire. The main dimensions that emerged were listening, confidence, empathy, decision-making, information and reassurance all listed at the end of the Table 2. The last step was to give coherence to the formulation of the questions such that the answers could be scored using a Likert-type scale from 1 to 4. Sometimes, and only if necessary, this involved expanding the number of response options to four. Then, the final questionnaire was translated into French, and translated back to English by a different bilingual person to verify that there had been no shift in meaning. The first version of the questionnaire was tested in a pilot study on the first 20 patients consulting one of a group of primary care physicians, whatever the pathology diagnosed. Immediately after the consultation patients were asked open questions as to whether they had understood the questionnaire and whether they had any remarks.

**Table 2:** Scales found assessing DPC in diverse conditions and settings.

Tool	Year and country	N° of items	Rating	Context	Domains studied	DPC-15 Question*	Source /reference
*CAHPS*: *Consumer Assessment of Healthcare Providers and Systems*	2008 USA, UK	95	4-point scale	Primary care	Quality of care communication	1, 2, 3, 4, 6, 7, 8, 11, 12, 14, 15	AHRQ Atlas https://www.cahps.ahrg.gov
*ACES*: *Ambulatory Care Experiences Survey*	2002 USA	34		Primary care	Quality of care: Doctor-patient interaction Organization of care	1, 6, 8, 9	AHRQ Atlas
*CARE measure*^*1*^	2004 UK	10	Likert: 1 to 5 NA	Primary care	Empathy	1, 2, 3, 5, 6, 7, 8, 15	Mercer (2004; Neumann (2012)
*Instrument for measuring patient-reported psychosocial care by physicians*^*1*^	2009 Germany	13	1	Hospital care	Support Information Shared decision making	1, 6, 8, 10, 11, 12, 15	Ommen (2009)
*HCS*: *Human Connection Scale*	2009 USA	16	Likert: 1 to 4	Hospital care Chronic disease Cancers	Therapeutic alliance	1, 3, 6, 10, 11, 12, 13, 15	Mack (2009)
*PCAS*: *Primary Care Assessment Survey*	1998 USA	51	Likert	Primary care	Performance of GP Satisfaction	1, 3, 7, 10, 12, 13, 15	Ingersoll (2005)
*Jefferson Scale of Patient’s Perceptions of Physician Empathy*	2007 USA	5	Likert 1 to 5	Hospital care	Empathy	1, 5, 15	Kane (2007)
*Patient perception of Quality*	2000 Canada	22	Likert 1 to 5	Primary care	Doctor-patient relation Technical aspects of care Result of the consultation	1, 3, 4, 6, 7, 8, 9, 10, 15	Haddad (2000)
*Patient Doctor Depth of Relationship Scale*	2011 UK	8	1 to 5	Primary care	Depth of relationship	10, 11, 15	Ridd (2011)
*Physician-Patient Concordance*	2004 USA, NZ	6	Likert: 1 to 4	Primary care	Agreement between doctor and patient concerning proposed care	5, 9, 15	Kerse (2004)
*PCAT*: *Primary Care Assessment Tool*	2001 USA	92		Primary care	Quality of care	2, 4, 6, 10, 12, 13	AHRQ Atlas
*Family-Centered Care Self-Assessment Tool*	2008USA	98	Likert 1 to 4	Primary care	Quality of care	3, 7, 9, 12, 13, 14	AHRQ Atlas
*Trust in Physician Scale*	2003 USA	11	Likert 1 to 5	Hospital care Chronic disease	Confidence	10, 11, 12, 13, 15	Freburger (2003)
*PC*: *Patient Perception of Continuity Instrument*	1988 USA	23	Likert 1 to 5		Continuation of care; Doctor-patient relation	7, 8, 10, 11, 12	AHRQ Atlas
*CPS*: *Control Preference Scale*	2012 USA	1	5 possible replies	Hospital care Chronic disease (Prostate cancer)	Shared decision making	9	Henrikson. (2012)
*SDM-Q-9*	2011 Germany	9	Likert 1 to 6	Primary care	Shared decision making	6, 7, 8, 9, 12, 14	Kriston (2010)
*PPOC*: *Patient Perceptions of Care*	2002 USA	40		Primary care	Communication Continuation of care	1, 3, 6, 7, 10, 12, 13	AHRQ Atlas
*PACIC*: *Patient Assessment of Chronic Illness Care*	2005 USA	20	Likert 1 to 5	Primary care Chronic disease	Communication Shared decision making Coordination of care	8, 9	AHRQ Atlas
*PPE-15*: *Picker Patient Experience*	2002 UK, Germany Sweden, Switzer-land, USA	15		Hospital care	Information Continuation of care Coordination of care	6, 9, 3, 10, 15	AHRQ Atlas
*QQPPI*: *Questionnaire on the Quality of Physician–Patient Interaction*	2010 Germany	14	Likert: 1 to 5	Primary care, ambulatory care	Communication Satisfaction	4, 6, 7, 8, 9, 11, 12, 15	Bieber, (2010)
*GP Patient Survey*	2013 UK	6	Likert 1 to 5 NA	Primary care ambulatory	Communication	1, 8,9, 12,15	NHS (2013)
*CPCQ*: *Client Perception of Coordination Questionnaire*	2003 Australia	31	Likert: 1 to 5	Primary care Chronic disease	Coordination of care Communication	9, 13	AHRQ Atlas

*Dimension explored by each question:

1 Listening, 2 Patience / respect for patient, 3 Attentiveness, 4 Physical Examination, 5 Empathy, 6 Clarity, 7 Completeness, 8 Disadvantages, 9 Decisional involvement, 10 Reassurance, 11 Privacy -> 2 respect,12 Confidence, 13 Truth -> 2 respect, 14 Understanding, 15 Concerns.

Questions in DPC-15. Questions 11 and 13 were found to be redundant by the validation study and were combined with question 2 in the final questionnaire DPCQ.

### Validation of the questionnaire

#### Sample size calculation

Using Confirmatory Factor Analyses and rule-of-thumb, estimates vary from 4 to 10 subjects per variable, with a minimum of 100 subjects to ensure stability of the variance–covariance matrix [29]. Thus, we set the requirement to 10 subjects per item. Since the DPC scale consists of 15 items, this meant the minimal sample size for the purposes of our study was 150. Allowing for 20% of patients potentially lost to follow-up we required 180 patients in total. We stopped the inclusions when the number needed was reached.

#### Design

A two-center prospective observational study was conducted from November 2013 to May 2014 in the emergency departments of two hospitals. The study was approved by the regional ethics committee of Clinical Investigation Centers (Rhône Alpes-Auvergne, Clermont Ferrand, Institutional Review Board n°5891 on 31-Oct-2013).

All consenting adults and adolescents (>15 and accompanied by a parent who gave their consent) diagnosed with either an ankle sprain or an acute infectious disease (infectious colitis, pyelonephritis, diverticulitis, prostatitis or pneumonitis) were informed of the study during the consultation, both by the physician and in a patient information letter. We chose these conditions as they are very frequent and are representative of the non-severe trauma and acute infectious diseases seen both in general practice and in emergency departments. The choice of emergency setting enabled us to avoid situations where the doctor already knew the patient. This also helped to maintain some homogeneity in the type of consultation, with physicians who had to work with a high and rapid turnover of patients and did not have the opportunity to improve DPC by prolonging the consultation [20,30]. We excluded patients whose consultation led to hospitalization for more than 48 hours because their care would be managed by the hospital with several doctors caring for them. Patients gave written informed consent to be contacted one week later and if the patient declined to participate, this was recorded in a log. The doctor who saw the patient then included them in the study by completing a short inclusion-case report form describing the patient’s baseline characteristics.

Patients were contacted by telephone 7 to 10 days after the consultation by a doctor who had not participated in patient recruitment. They were asked the series of generic questions on their perception of DPC during the consultation; the amount of information they had received, their adherence to treatment and/or the doctor’s recommendations. Patient data and answers were anonymized before double entry in an Excel database.

#### Statistical analysis

Statistical analysis was performed using Stata Version 13.0 (Stata Corp, College Station, Texas) software for OSX. Categorical variables are expressed as numbers and percentages and continuous variables as the medians and interquartile range (IQR) [25th and 75th percentiles]. To evaluate our DPC score, the consistency of the items was assessed by Cronbach’s alpha, giving an index between 0 and 1, followed by an unrotated Principal Component Analysis (PCA). The limitations of Cronbach’s alpha have been debated [31]. The main issue underlined by Sijtma is that the coefficient underestimates the reliability of a questionnaire, which was not a restriction for our purpose [31]. In psychometrics studies, PCA is often rotated in order to better understand and interpret component loadings. Nevertheless, this was not the purpose of our study, which is why we used an unrotated PCA.

## Results

### The DPC questionnaire

Our literature search lead to 22 selected scales (Fig 1). The dimensions they covered are listed in Table 2. The final questionnaire contained 15 items see Table 3 with 4 possible answers: no, possibly no, possibly yes, and yes, rated according to a Likert-type scale (1 to 4 points) to avoid the non-committal response inherent to 5 possible replies. The fifteen-item questionnaire (DPC 15) tested in the pilot study was well understood and took about 3 minutes to complete.

**Table 3:** Doctor-patient communication questionnaire.

QUESTIONS	No	Possi—bly no	Possi-bly yes	Yes
1. Did the doctor listen to you carefully during the consultation?	1	2	3	4
2. Did the doctor allow you to talk without interrupting you?	1	2	3	4
3. Did the doctor encourage you to express yourself / talk?	1	2	3	4
4. Did the doctor examine you thoroughly?	1	2	3	4
5. Do you feel that the doctor understood you?	1	2	3	4
6. Was it easy to understand what the doctor said?	1	2	3	4
7. Do you feel you were given all the necessary information?	1	2	3	4
8. Did the doctor explain the advantages and disadvantages of the treatment or care strategy?	1	2	3	4
9. Did the doctor involve you in the decision-making?	1	2	3	4
10. In your opinion, did the doctor have a reassuring attitude and way of talking?	1	2	3	4
11. *Do you think the doctor was in general respectful*?*	1	2	3	4
12. Did the doctor make sure that you understood his explanations and instructions?	1	2	3	4
13. *Do you think the doctor told the whole truth*?*	1	2	3	4
14. Do you have confidence in this doctor?	1	2	3	4
15. Did the doctor reply to all your expectations and concerns?	1	2	3	4

Total: /60

*Questions deleted following the validation study

Note: The questionnaire was constructed and validated in French. The French version has been translated into English and then back-translated into French by an independent translator to verify the conformity of the translation. The French version is available on request to the corresponding author.

### Clinical study

#### Population

**Fig 3:**
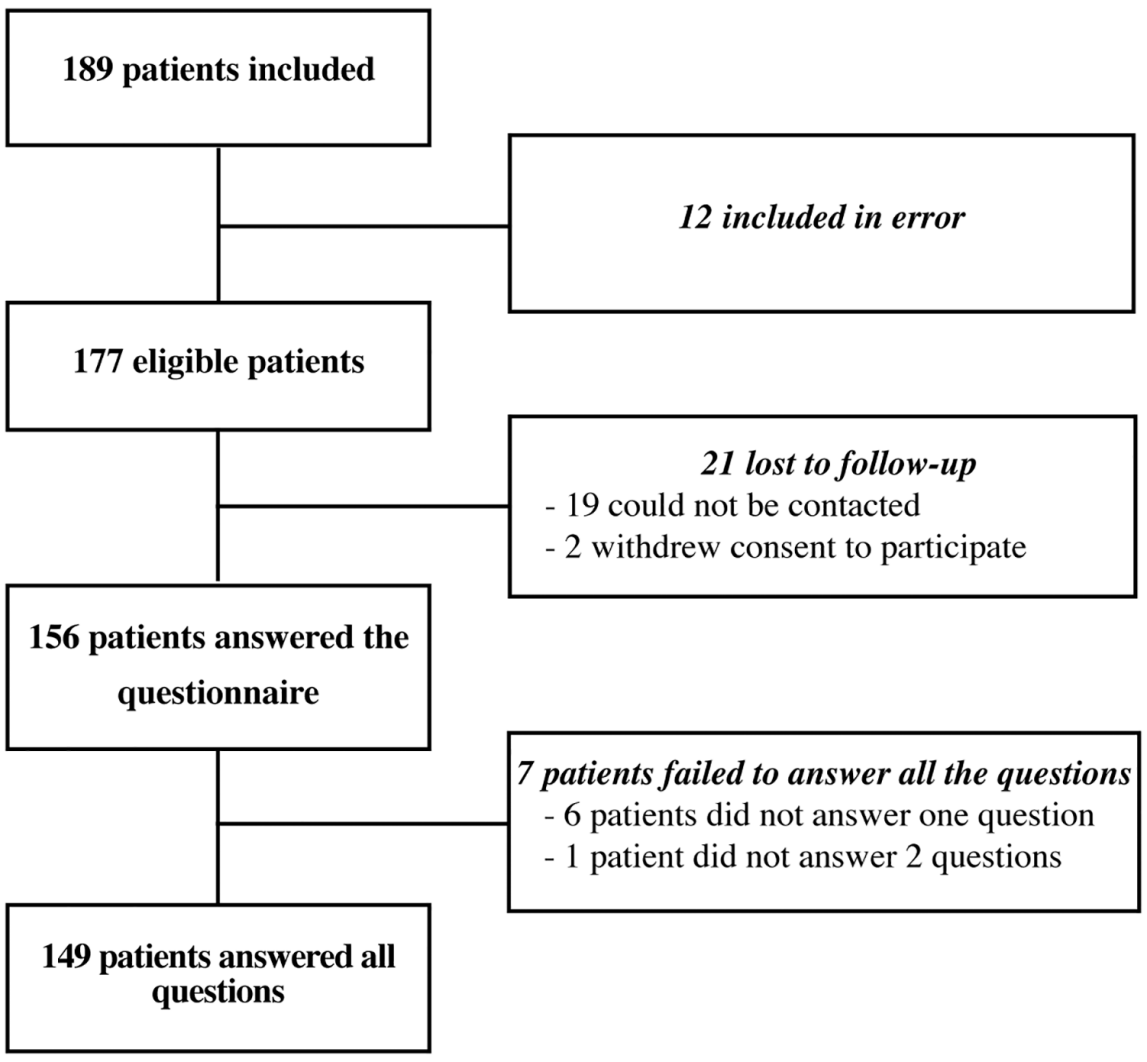
Flow chart of the population studied.

We included 189 patients. Of these patients, 12 were erroneously included (underage, hospitalization > 48 hours) and 21 were lost to follow-up (12%). Data from 156 patients were analyzed and 149 patients answered all questions. The patients were divided into two groups according to the diagnosis: ankle sprain: 83 patients (53.2%) and infection: 73 patients (46.8%) (Fig 3). Table 4 shows the characteristics of the patients and the median score of DPC-13 and DPC-15 questionnaires. The high level of response indicates the feasibility of use of the questionnaire.

**Table 4:** Characteristics of patients included in the validation study and DPC score values.

	All patients	Ankle sprain	Infection	P-value
	n = 156	n = 83	n = 73	
**Male, n (%)**	61 (39.1)	42 (50.6)	19 (26.0)	0.002
**Age, median (IQR)**	36 (23–57)	27 (20–39)	52 (30–68)	< 0.001
**Age groups, n (%)**				< 0.001
<40 years	85 (54.5)	63 (75.9)	22 (30.1)	
≥40 years	71 (45.5)	20 (24.1)	51 (69.9)	
**Education completed, n (%)**				0.03
Secondary school	62 (29.7)	25 (30.1)	37 (50.7)	
High school	39 (25.0)	25 (30.1)	14 (19.2)	
University	55 (35.3)	33 (39.8)	22 (30.1)	
**Worked in a medical setting, n (%)**	23 (14.7)	11 (13.3)	12 (16.4)	0.6
**Family status, n (%)**				0.1
Single	79 (50.6)	47 (56.6)	32 (43.8)	
Couple	77 (49.4)	36 (43.4)	41 (56.2)	
**DPC-15 questionnaire (/60)** *	**n = 149**	**n = 81**	**n = 68**	0.2
median (IQR)	52 (46–56)	52 (47–57)	52 (46–55)	
mean (SD)	49.8 (8.9)	50.1 (9.4)	49.4 (8.4)	
**DPC-13 questionnaire (/52)**				0.2
median (IQR)	44 (39–48)	44 (39–49)	44 (39–47)	
mean (SD)	42.0 (8.6)	42.3 (9.2)	41.8 (7.9)	

IQR: Interquartile range

* Principal Component Analysis was performed on 149 patients due to 7 incomplete questionnaires (6 patients didn’t answer to one question and 1 patient didn’t answer 2 questions).

#### Internal and external validity of the DPC questionnaire

Cronbach’s alpha was calculated for 149 of the 156 patients, because 7 patients did not answer all the questions. Alpha was calculated as 0.89, indicating good internal consistency of the questionnaire (> 0.8). The correlation coefficients for items 11 ("Do you think the doctor was in general respectful?"), 13 ("Do you think the doctor told the whole truth?") and 2 (“Did the doctor allow you to talk without interrupting you?”) were weaker (r<0.45) see Table 5.

**Table 5:** Internal coherence of the scale: Correlation score and Cronbach’s alpha score for each item.

Item	Number of patients	Sign	Item-test correlation	Item-retest correlation	Average Inter-item covariance	Cronbach’s alpha
**dpc-1**	156	+	0.74	0.70	0.32	0.87
**dpc-2**	153	+	0.44	0.37	0.34	0.89
**dpc-3**	155	+	0.69	0.61	0.30	0.88
**dpc-4**	156	+	0.59	0.51	0.32	0.88
**dpc-5**	155	+	0.77	0.72	0.31	0.87
**dpc-6**	156	+	0.58	0.53	0.34	0.88
**dpc-7**	156	+	0.81	0.76	0.29	0.87
**dpc-8**	156	+	0.62	0.51	0.31	0.88
**dpc-9**	155	+	0.47	0.35	0.33	0.89
**dpc-10**	156	+	0.57	0.49	0.33	0.88
dpc-11*	**156**	**+**	**0.39**	**0.35**	**0.36**	**0.89**
**dpc-12**	156	+	0.70	0.65	0.32	0.88
dpc-13*	**156**	**+**	**0.37**	**0.33**	**0.36**	**0.89**
**dpc-14**	154	+	0.77	0.71	0.30	0.87
**dpc-15**	156	+	0.84	0.80	0.29	0.87
**Test score**	1	2	2.3	2.4	0.32	0.89

**dpc**: doctor-patient communication question

* Redundant questions were deleted from the final questionnaire.

**Fig 4:**
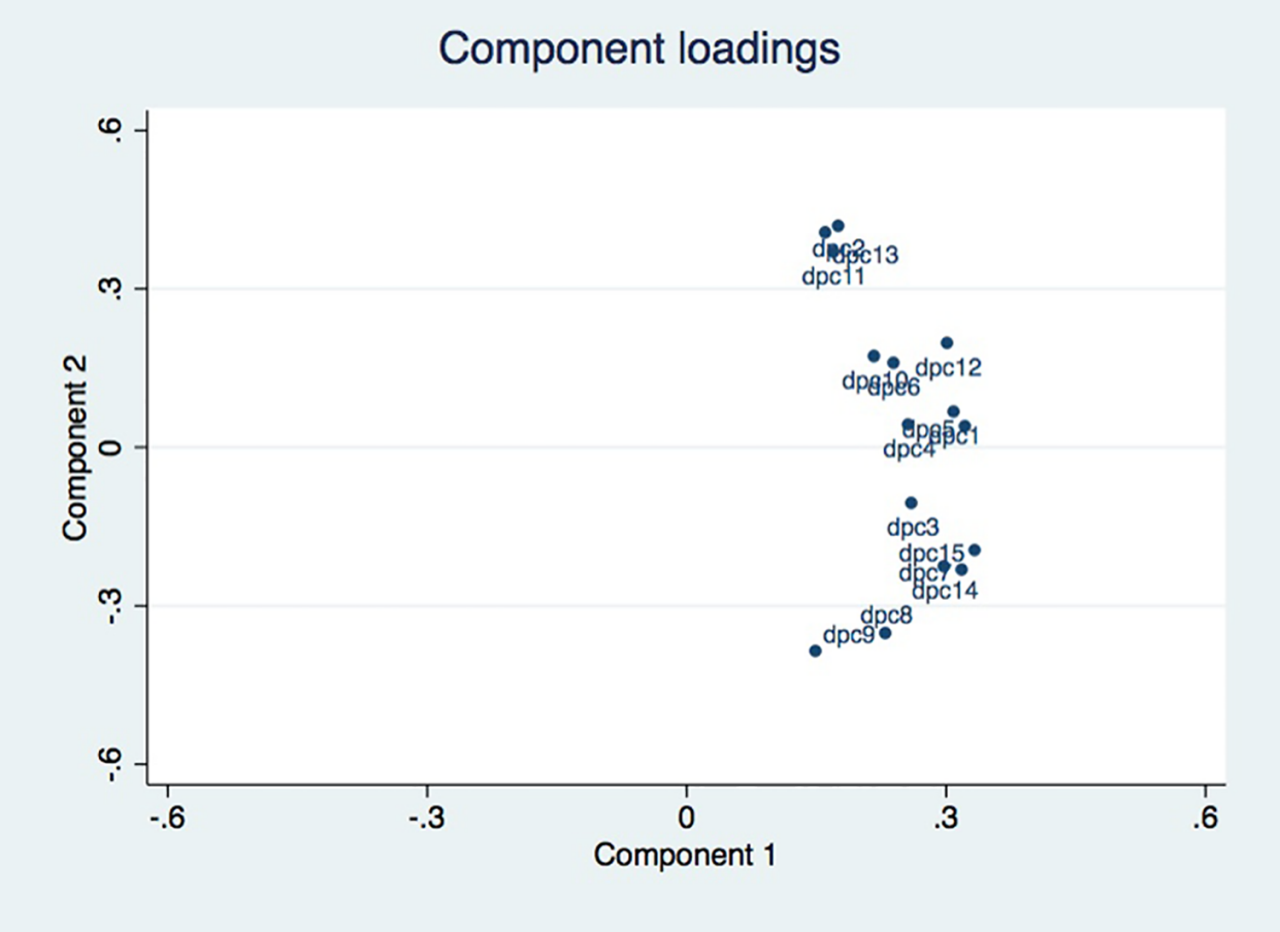
Principal Component Analysis (PCA) on DPC-15.

The Principal Component Analysis showed a "butterfly wing" distribution of items for Component 2, showing that the items were exploring the same dimension. Fig 4 shows the PCA. Its two main components are: component 1, with an eigen value of 6.2 and a proportion of explained variance of 0.42; and Component 2 with an eigen value of 1.4 and an explained variance of 0.10. Supporting information S1 Table presents the contribution of each variable to the two main components of the score.

From the statistical results, and after discussion with a multidisciplinary committee, items 11 and 13 of “DPC 15”, often misunderstood by patients, with low coefficients of correlation and redundant in the principal component analysis with item 2 ("Did the doctor stop you while you were talking?") were removed in the final version. The final scale thus includes 13 questions each rated from 1 to 4: DPC-13.

For external validity, DPC is one of the determinants of adherence to treatment [32] and we found a correlation between DPC and adherence (article submitted).

## Discussion

### Characteristics of the new DPC scale

The new scale we developed has several advantages: 1) it is concise (time required to answer is 3 minutes on average) which is important for research, particularly in an emergency setting (13 items compared to 98 in scales such as the "Family-Centered Care Self-Assessment Tool"), 2) the closed answers make it easy to use, 3) it is independent of the acute condition, 4) it results from a multidisciplinary collaboration and 5) it is based on a theoretical model. The DPC-scale had good internal coherence [33].

The median score for DPC-15 was 52/60 reflecting positive results. At a later stage it will be important to determine whether these good results are related to the particular hospital, to the type of clinical situations studied (acute diseases) or to the DPC itself, and in what proportions.

The high participation rate of patients confirmed that this scale was easy to use and well accepted. The percentage of patients lost to follow-up was low (12%). Only two patients refused to participate and none interrupted the telephone interview. We should mention that information as to whether the patient is seen alone or accompanied should be noted as this may interfere with DPC [23].

### Strengths

Although we started from a theoretical model to facilitate our choices (inclusion/exclusion) of items [28], our premise was to consider the exhaustive list of items characterizing DPC coming from 30 years of research in medicine, pharmacy and psychology. Thus by engaging a multidisciplinary team of physicians, pharmacists and health psychologists to make a synthesis of previous work it was possible to build a generic scale that respected our inclusion criteria without omitting any essential concepts. Although only articles in English were included, we believe our literature search covered all main aspects, as confirmed by comparison with literature reviews on the subject and the considerable work of the AHRQ [34]. The scales were from many countries (United States, Switzerland, Australia, and the UK) and cover a period of 30 years of research (the oldest listed scale was from 1988). Furthermore, we achieved saturation after analyzing the 11 tools in the “AHRQ Atlas”; further searches failing to augment the number of relevant items. Thus we consider what we found as representative of the diverse aspects of DPC.

### Comparison with other scales

The main differences between this and other scales concern both the methodological choices used to develop the scale (based both on a synthesis of the literature and on a theoretical model) and/or also in the items selected.

Although the development process for the Communication Assessment Tool (CAT) was similar to ours, with a review of frequently used models and tools, it was developed in the context of teaching and assessing physicians’ and medical students’ communication skills, which was one of our exclusion criteria [24]. We compared the scale we had derived from the literature review (DPC 15) to the CAT scale. Our DPC scale covered all of the CAT items, but the CAT does not cover all of our items. Furthermore, according to a the literature^13^ some important items are missing in the CAT (e.g. "Did the doctor involve you in the decision-making?" “Do you have confidence in the doctor?”).

Although consultation length is a key feature of many DPC scales, such as the GPPS (“the doctor giving you enough time”), the CAT (“the doctor spent the right amount of time with me” or the QQPPI questionnaire [25] (“the doctor spent sufficient time on my consultation”), we decided to exclude these items, as they seem inappropriate for acute conditions. Even though the doctor may have “spent enough time with the patient” they may not have responded to the patient’s communication expectations. Moreover, a very recent study points out that there is no association between consultation length and patient experience of communication [35,36]. It suggests that we should no longer include items related to the perception of time spent in consultation in DPC scales. Similarly, items such as eye contact weren’t included as this is difficult to measure without video recording the consultation and difficult to assess by patient self-reporting. To keep the questionnaire short and easy to use, we restricted items to those directly related to DPC and did not include organizational aspects.

Overall, despite some similarities between our scale and those developed by other authors such as Makoul (CAT) or Bieber (QQPPI), existing scales did not meet our requirements and justify the development of a scale for acute conditions.

A recent study proposes a new general scale for assessing tools such as PILs [37]. However, it does not attempt to measure doctor-patient communication specifically, but a broader range of outcomes in the same time (emotional, cognitive and behavioral), possibly because the elaboration of this scale was not based on any preliminary theoretical model.

### Limitations

All the tools we extracted from the “AHRQ Atlas”[34], although validated, were not found by our systematic review. The field of DPC is extremely broad extending over several disciplines, with several thousands of articles published. This obliged us to use filters in our literature searches, which may have restricted exhaustivity.

Regarding our selection of tools, it seemed appropriate to take the patient’s point of view when evaluating DPC [30,38,39]. A patient’s experience will more directly predict their behavior (adherence, compliance with given advice) than the doctor’s point of view [39,40]. Self-evaluation is frequently used in clinical practice as it gives more reproducible results, is more reliable, less expensive and less invasive than direct measurements [22,28,37,38,41]. However, it would be interesting to explore the physician’s point of view about DPC in a further study, since the expectations and perceptions of the patient and of the physician may be very different [42,43].

The study coordinator received no non-inclusion forms. This may be explained by the lack of time in ED. Moreover, it is possible that patients lost to follow-up (who could not be contacted by telephone after 3 attempts) were the ones least satisfied with the consultation. This inclusion bias may lead to an over-estimated of results.

To limit the subjectivity biases, the investigator who assessed DPC was independent of the emergency department.

For reasons of feasibility, we limited our study to 6 AC corresponding to 2 subgroups of pathology (trauma versus infection) that are very different clinical situations in age and sex (ankle sprain in the young man and infection in older woman). The heterogeneity of the population, representative of these two clinical situations, does not affect the DPC scores that are comparable for the two subgroups, for the response rates and for the feasibility of the study. Nevertheless, these two clinical situations do not represent the totality of the clinical situations and the patient profiles encountered in the ED or primary care setting and it would be interesting in future works to extend the research to other clinical situations and / or populations.

## Conclusion

This new DPC scale could open new perspectives in clinical research for assessing the impact of any tools aimed at improving DPC during a consultation for acute conditions, where patients are the first to suffer from a lack of information [28]. This tool could be valuable to the research community and enable the results of different studies to be compared, quantitate DPC and correlate its level with other outcomes such as patients’ adherence to treatment, knowledge and/or satisfaction in the particular context of acute conditions.

## Supporting information

S1 Table The contribution of each variable to the two principal components of the score. (DOCX)

S2 Table Raw validation study data. (XLSX)

S1 File Protocol in French. (DOCX)

S2 File PRISMA checklist. (DOC)
