# Supplementary material for: A scale assessing doctor-patient communication in a context of acute conditions based on a systematic review
Source: PLoS One. 2018 Feb 21;13(2):e0192306. doi: 10.1371/journal.pone.0192306 (PMC5821327; doi:10.1371/journal.pone.0192306)
Supplement: S1 Table — (DOCX) [file pone.0192306.s001.docx]

--------------------------------------------------------------------------

Component | Eigenvalue Difference Proportion Cumulative

-------------+------------------------------------------------------------

Comp1 | 6.21715 4.78605 0.4145 0.4145

Comp2 | 1.4311 .251573 0.0954 0.5099

-------------+------------------------------------------------------------

------------------------------------

Variable | Comp1 Comp2

-------------+----------------------

DPQ 1 | 0.3106 0.0647

DPQ 2 | 0.1760 0.4176

DPQ 3 | 0.2620 -0.1081

DPQ 4 | 0.2580 0.0392

DPQ 5 | 0.3234 0.0381

DPQ 6 | 0.2399 0.1564

DPQ 7 | 0.3199 -0.2326

DPQ 8 | 0.2313 -0.3528

DPC 9 | 0.1512 -0.3871

DPC 10| 0.2182 0.1687

DPC 11| 0.1710 0.3668

DPQ 12| 0.3028 0.1930

DPQ 13| 0.1620 0.4037

DPQ 14| 0.2989 -0.2283

DPQ 15| 0.3352 -0.1963

------------------------------------
