# Supplementary material for: A scale assessing doctor-patient communication in a context of acute conditions based on a systematic review
Source: PLoS One. 2018 Feb 21;13(2):e0192306. doi: 10.1371/journal.pone.0192306 (PMC5821327; doi:10.1371/journal.pone.0192306)
Supplement: S1 File — (DOCX) [file pone.0192306.s003.docx]

**PROTOCOL**

**GENERAL INFORMATIONS**

| **Title :** An inventory of levels of Doctor-Patient Communication (DPC) during a consultation for acute condition (AC) in Emergencies Departments (ED) in order to improve practices. Impact on overall adherence and patient satisfaction for ankle sprain or acute pyelonephritis in two EDs. |
| --- |
| **Methodoldoctor-patient communicationy:** Prospective, multicenter, observational study using declarative data |
| **Coordinating investigators authorized to sign protocol:** Dr Mélanie SUSTERSIC, Pr Jean-Luc BOSSON |
| **Coordinating Investigators:** Anais Kernou, Charlotte Gibert, internes en Médecine Générale |
| **Investigators:** Emergency physicians (Emergency Department of the Clinique Mutualiste in Grenoble and Emergency Department of the Center Hospitalier de Chambéry) |
| **Experts :** Pr Jean-Luc BOSSON (CIC, TIMC IMAG), Dr Aurélie Gauchet (Laboratoire Inter universitaire de Psycholdoctor-patient communicationie, UPMF) |
| **Study Coordinating Center : CIC**  Centre d’Investigation Clinique – Inserm003  CHU de Grenoble, 38043 Grenoble Cedex 09  Tel : 04 76 76 92 60 Fax : 04 76 76 92 62 |

| **PROTOCOL– SUMMARY** |
| --- |
| **Coordinating investigators authorized to sign protocol** Mélanie SUSTERSIC, Pr Jean-Luc BOSSON |
| **Coordinating Investigators**: Anais Kernou, Charlotte Gibert |
| **Investigators**:Médecins Urgentistes (Service d’Urgences de la Clinique Mutualiste à Grenoble et Service d’Urgences du Centre Hospitalier de Chambéry) |
| **Investigators**:Pr. Jean-Luc BOSSON, Dr Aurélie GAUCHET |
| **Methodoldoctor-patient communicationy:** Prospective multicenter observational study |
| **Main objective:** To assess the state of DPC levels, overall adherence (GASAC) and patient satisfaction in two emergency departments to improve practices.  **Primary outcome:** Measurement of DPC, GASCA scores and patient satisfaction between D7 and D10 following a consultation in two emergency departments (for 2 frequent reasons: ankle sprain and acute pyelonephritis). |
| **Secondary objectives:**  1. Evaluate the psychometric properties of the DPC and GASAC scores created for the study;  2. To evaluate the correlation between Moriskys therapeutic adherence and the "drug adherence" dimension of the GASAC score created for the study;  3. Evaluate the correlation between Morisky's adherence and patients' GASAC;  4. Evaluate the correlation between DPC and GASAC;  5. Assess the correlation between DPC and satisfaction;  6. Assess the correlation between GASAC and satisfaction.  **Secondary outcomes:**  1. Measurement of the psychometric properties (Cronbach's coefficient) of DPC and GASAC scores. The 2 scores will be measured in a telephone survey between D7 and D10 after the consultation.  2. Measurement of the correlation between Morisky Green's therapeutic adherence score and the "drug adherence" sub-dimension of the GASAC score created for the study;  3. Measurement of the correlation between the Morisky Green compliance score and the GASAC score;  4. Measurement of the correlation between DPC score and GASAC score;  5. Measurement of the correlation between DPC score and satisfaction score;  6. Measurement of the correlation between the score of GASAC and the score of satisfaction. |
| **Main Inclusion Criteria:** major, or legal guardian accompanying a minor aged 15 years and 3 months or more, literate, having consulted in one of the emergency departments for one of the selected patholdoctor-patient communicationies (ankle sprain or acute pyelonephritis) and can be contacted by telephone ten days. |
| **Total number of patients to include:** 200 to obtain sufficient accuracy in the descriptive study of the main objectives (95% CI of the order of 5%) |
| **Total duration of the study:** 4 months |
| **Duration of the study per patient:** 7 to 10 days |

**JUSTIFICATION OF THE STUDY, GENERAL CONTEXT**

I. The HPST law (Act No. 2009-879 of 21 July 2009 on Hospital, Patient, Health and Territory Reform) (1) establishes the obligation of continuing professional development (CPD) for health professionals. According to section 59 of the Act, the CPD's objectives are "the evaluation of professional practices, the improvement of knowledge, the improvement of the quality and safety of care as well as the consideration of public health priorities and the medical control of health expenditure ".

In particular, the Order of 26 February 2013 sets out two specific national guidelines for 2013:

- "contribute to the improvement of patient care" (notably by "knowledge of the health status of the patient" and "clinical and epidemioldoctor-patient communicationical studies prdoctor-patient communicationrams aimed at evaluating practices"

- "contribute to the improvement of the relationship between healthcare professionals and patients" (notably through "the development of information and patient autonomy;" and with CPDs aiming at " compliance with treatments ", as well as" training in the professional health-patient relationship or in the care-and-care partnership. ")

It is not surprising that these two concepts of therapeutic adherence and physician-patient communication (DPC) are on the agenda of national public health objectives.

Indeed, according to Haynes, "in terms of EO, it is defined by the extent to which an individual's behaviors (in terms of medication, diet follow-up or lifestyle changes) coincide with medical or health advice "(3). The evaluation of adherence has been the subject of many recent publications. Improving adherence is one of the main actions to achieve one of the main goals of caregivers: improving the health and quality of life of patients (4). For WHO, "Optimizing drug adherence would have more impact in terms of global health than the development of new drugs" (5).The importance of EO was emphasized, particularly by WHO, for chronic diseases mainly (6). Whether it is acute or chronic patholdoctor-patient communicationy, it is essential that all patients understand and know the patholdoctor-patient communicationy and the treatment that leads them to consult (7,8,9). In addition, "non-compliance" has many medical and economic consequences (4,10).Compliance is therefore an important dimension that reflects the patient's behavior in terms of follow-up of medicinal prescriptions and health and dietary advice and is thus a reflection of the quality of a consultation.

However, observance is an "unstable, dynamic and modifiable phenomenon" (11). Many influencing factors have been identified. The WHO lists 5 classes, including those related to the health care system that include the quality of the therapeutic relationship (5). Similarly, for many health psycholdoctor-patient communicationists, (12) it is doctor-patient communication during the consultation that is crucial in the formation of adherence or non-compliance (11). Indeed different parameters take into account: the delivery of information and the understanding of it by the patient as well as the affective dimension of the relationship. "The feeling of being understood and informed improves compliance" (13).Poor therapeutic relationships would be a factor of non-adhesion (4,14,15,16).

Being able to evaluate these two parameters (OT and DPC) easily, therefore seems to us crucial in a CPD approach, aiming ultimately for their improvement.

However, there is no generic score in the literature evaluating doctor-patient communication or therapeutic adherence, which may be comparable from one patholdoctor-patient communicationy to another. Two generic scores, one of DPC and one of global observance (DOCTOR-PATIENT COMMUNICATION), were therefore created for this purpose by the same research team during a preparatory phase (details in the protocol).

Our study will take place in 2 phases of which only the first phase is the subject of this protocol:

- A first phase (protocol below) with the objective of making an inventory of doctor-patient communication (DPC) levels, general observance (GA) and patient satisfaction in a health service, emergencies to improve practices; and on the other hand, the evaluation of the intrinsic qualities of these 2 scores (Cronbach's coefficient). These two scores are intended to be used as an indicator of the quality of professional practices for any clinical situation.

At the same time, we will evaluate the correlations between GA, DPC and baseline patient satisfaction in an emergency department. Indeed, if this link exists, any prdoctor-patient communicationram aimed at improving

**I. OBJECTIVE OF THE STUDY**

- 1. **Primary objective :**

Conduct an inventory of physician-patient communication (DPC) levels, overall compliance DPC and patient satisfaction in two emergency departments to improve practices.

**Critère de jugement principal :**

Measurement of CPD score, DPC and patient satisfaction following a consultation in two emergency departments (for ankle sprain and acute pyelonephritis). The 2 scores will be measured in a telephone survey between D7 and D10 after the consultation.

**I.2. Secondary objectives:**

1. Evaluate the psychometric properties of the DPC and DOCTOR-PATIENT COMMUNICATION scores created for the study;

2. To evaluate the correlation between Morisky Green's therapeutic adherence and the "drug adherence" sub-dimension of the DOCTOR-PATIENT COMMUNICATION score created for the study;

3. Evaluate the correlation between Morisky's adherence and the patient's DOCTOR-PATIENT;

4. Evaluate the correlation between CPD and DOCTOR-PATIENT COMMUNICATION;

5. Evaluate the correlation between CPD and satisfaction;

6. Evaluate the correlation between DOCTOR-PATIENT COMMUNICATION and satisfaction.

**Secondary outcomes :**

1. Measurement of the psychometric properties (Cronbach's coefficient) of the DPC and DOCTOR-PATIENT COMMUNICATION scores.

2. Measurement of the correlation between Morisky Green's therapeutic adherence score and the medication adherence sub-dimension of the DOCTOR-PATIENT COMMUNICATION score created for the study;

3. Measurement of the correlation between the Morisky Green compliance score and the DOCTOR-PATIENT COMMUNICATION score;

4. Measurement of the correlation between the CPD score and the DOCTOR-PATIENT COMMUNICATION score;

5. Measurement of the correlation between the CPD score and the satisfaction score;

6. Measurement of the correlation between the DOCTOR-PATIENT COMMUNICATION score and the satisfaction score.

1. **DESIGN OF RESEARCH**

**Study design**

• Observational study

• Multi-center study

• Prospective study using declarative data

**General oragnization of the study**

**Experimental Scheme**

Survey by telephone questionnaire with single measurement on D7-10 for the two clinical situations.

**Conduct of the study**

***Preparatory phase for the development of the questionnaires***

In order to evaluate therapeutic adherence as well as DPC, with scores that can be compared from one pathology to another, we created, in a first phase, generic adherence scores and CPD scores, common to all pathologies (and not a specific score for each pathology, as it exists in the literature).

*The GASAC score (Appendix 1)*

It includes 2 main parts:

*• The first part A / of the questionnaire corresponds to the therapeutic compliance score of Morisky-Grenn (23)*

It is a validated score, adapted to the French language and used for high blood pressure, widely used (it is the reference in chronic pathologies and is online on the site of the Social Security to allow doctors to assess patient compliance).

This score was not used in an acute situation in its initial version. It was therefore first modified by adapting the questions in the context of the acute situation by a select committee of experts (Pr JL Bosson, Dr M Sustersic, Dr A Gauchet). The score remains unchanged with the same 6 questions and definition of a non-observing patient (score ≥ 3).

*• The second part B / corresponds to the newly created CPD score.*Since there is no generic observation score for acute pathologies in the literature and no evaluative score for both the drug and non-drug aspects of the observation for acute pathologists, A new composite score. We used several of the questionnaires not a validated score used in the evaluation of adherence to HIV treatment by anti-retrovirals (13), SATMED-Q (24) and AHRQ questionnaires ( 25).

We will call the newly created observation score, Compliance Compliance Score (CPAD) as opposed to the Morisky Green drug observation score or the definitive TO concept. This DPC score includes 4 sub-dimensions:

- adherence to drug prescriptions (same target as the Morisky Green score);

- adherence to non-medicinal prescriptions (supplementary examinations);

- compliance with hygiene-dietetic rules;

- behavior in terms of health care (reconsultations ...).

We measure the score of Morisky-Green in the first part, in order to rely on a

validated score. Indeed, if this second part, that is to say our newly created CPD

score, is correlated to the first part, we can then use the CPD score autonomously,

without having to use the Morisky-Green score.

***The Doctor-Patient Communication score (Appendix 2):***

Its development was the subject of a preliminary study carried out by the same team of researchers (physicians, statisticians, health psychologists, pharmacists, medical interns and psychology students). A review of the literature on the various scores and scales was carried out. From these scales were extracted the relevant items to evaluate the CPD (Appendix 3: methodology for developing the CPD score), which resulted in a new CPD questionnaire (Appendix 2).

*The general questionnaire: data sociodemographic and satisfaction score (appendix 4)*

It was developed to eliminate certain biases due to socio-demographic data and to carry out subgroup analyzes.In addition, the analysis of patient satisfaction (in a comprehensive manner and on certain details of the care) during their passage to the emergency room is essential to allow the validation of changes in professional practices.This will seek correlation between satisfaction and DPC.

***Recruitment of Investigators***

Contact with the administrative staff and the Chief Medical Officer of the Emergency Department of the Clinique Mutualiste de Grenoble to present the project, as well as the administrative staff and the Chief Medical Officer of the Emergency Department of the Center Hospitalier de Chambéry.Then short training and delivery of an explanatory letter on the study to emergency physicians likely to participate in the inclusion of patients (Appendix 5).

***Patient Inclusion***

During the inclusion period, when a patient is referred for either an ankle sprain or acute pyelonephritis, the investigating physician will offer to participate in the study and provide the information and non-opposition form (Annex 6), explaining the course and interest of the project. If the physician agrees, physicians will complete an inclusion form (Appendix 7) for each patient that will be retrieved by the study coordinators on site within a maximum of 3 days. This sheet will contain all the elements of identification necessary for quality monitoring. These personal data will not be computerized; they will only be used for telephone follow-up and only the results will be anonymously included in the database.

In case of refusal to participate in the study, the refusal of participation box will be checked on the inclusion form which will be filled on the day of the consultation and also recovered by the coordinators within a maximum of 3 days following the consultation.

***Phone survey***

Between 7 and 10 days after the patient was consulted in the emergency room, the inter-doctor-patient communicated by telephone using the general questionnaire (Appendix 4) and the DPC (Appendix 1) questionnaires and DPC (Annex 2).

If he can not be contacted the first time, we will try to reach him again twice (we will have asked him beforehand, at the time of inclusion, on what time range he wishes to be contacted). As a last resort we will call one of his relatives (only if he / she is a trusted person within the meaning of the Public Health Code, previously identified by the patient before inclusion.) If the patient fails will be considered lost.

***Investigative procedures conducted and differences from usual careProcédures d’investigation menées et différences par rapport à la prise en charge habituelle***

No difference from usual care. Only telephone contact with the patient is expected. An information letter requesting authorization will be provided (Annex 6).

The patient's telephone call procedure between D7 and D10 is as follows:

- The appellant will present himself by name and will ask to speak to the participant, without specifying the reason for the appeal to a third party.

- Then he will introduce himself to the participant by name, assignment, and will refer to the study, after making sure that it is the patient himself.

***Synopsis***

| Preparatory phase of 6 months | Development of questionnaires  Contact and brief training of investigating physicians |
| --- | --- |
| J0 | Emergency consultation and patient inclusion |
| J0 à J7 | Patient return home |
| J7 à J10 | Patient call and telephone survey with elaborate questionnaire, data collection |
| M2-M4 | Statistical analysis of results and interpretation |

1. **POPULATION STUDIED**

**Modalities of recruitment of subjects**

Major patient, or legal guardian of a minor aged 15 years and 3 months or more, who consults with the Emergency Department of the Clinique Mutualiste de Grenoble for one of the following two pathologies: ankle sprain or acute pyelonephritis .

**Inclusion Criteria**

The subjects meeting each of the following criteria will be proposed for the study:

- Major Patient ;

- or legal guardian of a minor (aged 15 years and 3 months or more), consultant for the pathology of the child ;

- Patient can be contacted by phone within 7 to 10 days.

**Exclusion Criteria**

Subjects meeting at least one of the following criteria may not be included:

- Patient illiterate ;

- Patient refusing telephone follow-up ;

- Patient with a visual or hearing impairment ;

- Non-Francophone Patient ;

- Persons deprived of their liberty by judicial or administrative decision, a person subject to a legal protection measure.

1. **CHOICE OF PATHOLOGIES**

The number of 2 pathologies seems to be a good compromise between completeness (evaluation of 2 different acute clinical situations: one concerning traumatology and the other infectious medicine) and the feasibility of the study.

We have chosen two pathologies frequently encountered in emergency departments and for which the management and advice given are twofold: medicinal and non-medicinal (hygiene-dietetic rules and proposal for follow-up and supplementary examinations) that the two sides of therapeutic adherence can be evaluated.

**MEASURED VARIABLES AND METHODS OF MEASUREMENT**

Responses to the questionnaires during the telephone survey between D7 and D10 following the inclusion and calculation of the scores.

- Adherence score (appendix 1): The adherence score according to Morisky is rated from 0 to 5. The overall compliance score proposed by our team includes 4 sub-dimensions each rated from 1 to 4 according to a scale of Likert. The score being the median of the sub-dimensions.

- DPC score on 60 points (Annex 2),

- Measurement of intrinsic properties (Cronbach's coefficient) and extrinsic adherence and CPD scores

- Satisfaction score (Annex 4): calculated from questions 6 to 10 of the general questionnaire, in order to obtain a total score rated on 20 points (Likert scale from 1 to 4)

- Correlation between DPC score, GASAC score and satisfaction score.

1. **DATA COLLECTION AND MANAGEMENT**

**The personal data**

Information about the included patients will be collected by the investigator and noted on the inclusion form (Appendix 7): surname, first name and telephone contact.

For each patient, an identification code is constructed: it corresponds to the first 2 letters of his name followed by the first letter of his first name.

The coordinators will retrieve all the forms at the Clinique Mutualiste, within a maximum of 3 days following the consultation.

These will be used to contact patients and will be archived in a protocol-specific record, which will be the source data.

There will be no computerized processing of personal data.

Only patient identification codes will be used for computer processing of telephone survey data.

**The questionnaires**

The data are collected through a telephone survey conducted by the coordinators: Anais Kernou, Charlotte Gibert, Amélie Duvert and Laure Gonnet, general practitioners at the end of the cycle. The medium used corresponds to questionnaires (Annexes 1, 2, and 4) and will be completed during the telephone call.

1. **STATISTICAL ANALYSIS OF MEASURED PARAMETERS**

**Calculating the number of subjects**

The objective is to obtain a minimum of 75 completed questionnaires per pathology. Since we are testing 2 cards, we will need 150 questionnaires.

To obtain 150 questionnaires, with an estimated loss of 25%, it will take 200 patients or 100 per pathologies

**The analysts**

- Editor of the statistical analysis plan: Professor J-L Bosson

- Persons responsible for carrying out the analysis: coordinators, Anais Kernou, Charlotte Gibert, Mélanie Sustersic

**Location of data analysis**

Center of Clinical Investigation of the CHU of Grenoble.

The data will be entered via an ad hoc input interface. Quality control on 100% of the data and 10% of the individuals will be carried out.

The statistical analysis and archiving of the database (after the basic frost procedure) will be carried out under the responsibility of Professor Jean Luc Bosson. The statistical analysis will be done with the STATA Version 10 OSI (StataCorp LP 4905 Lakeway Drive College Statio, Texas 77845 USA).

**Data analysis method**

The descriptive analysis covers all the variables collected. It will use visual descriptors (number and frequency for quantitative, median and inter-quantity variables for continuous variables). The statistical correlation tests between scores will be done with the risk of error of the first common species alpha = 0.05. The main criteria will be measured with the calculation of the 95% confidence interval.

For each score an ACP and the calculation of the Cronbach coefficient will allow to characterize these scores.

1. **ESTIMATED CALENDAR OF THE STUDY**

• Duration of the preparatory phase: development of questionnaires: spring - summer 2013

• Duration of study per patient: 7 to 10 days

• Total duration of the study: 4 months

• Intended Inclusion Date: November 2013

• Intended end date: January 2013

• Expected date of completion of study: February 2014

• Estimated date for the secondary phase of the study (FIP impact on tge assessment of DPC): year 2014

1. **STUDY STOPPING RULES**

## Study stopping criteria for a participating subject

Participants will be excluded from the study and replaced:

- In case consent is withdrawn
- In case of violation of an inclusion criterium (particular attention will be given to TCDB criteria, pregnancy, and conditions possibility of return home).

A 24hour safety follow-up must be performed if the patient is discharged directly from the ED.

Included subjects who cannot be discharged and return home for any unplanned reason but meet all the inclusion criteria will not be excluded from the study.

## Early discontinuation of the experimental procedure by the subject

TCUS should not be abandoned in the TCUS group. In case of difficulties, investigators are asked to find an experienced member of staff to perform the examination. In case of doubts on the quality or interpretation, a dedicated email box will be created to help make decision making compliant with the protocol.

##

## Study stopped by the sponsor

The sponsor may stop the study at any time, for the following reasons:

- Inability of the investigator to include subjects according to the planned schedule.

- Absence of signed consent.

- Major violations of the protocol.

- Incomplete or incorrect data.

The sponsor must make a declaration of the discontinuation of the study within 90 days of stopping it:

- When the study has been stopped in France,

- or, if the study is multinational, when it has been stopped in all the countries concerned, whether within the European Community or not.

- If the stop (definitively) of a clinical trial is anticipated, its discontinuation must be declared to the health authorities (ANSM) within 15 days, stating the reason.

## Study stopped by the investigator

In the case of adverse events judged as severe by the investigator and that could jeopardize the health of subjects, the investigator may stop the study with the sponsor’s agreement.

# Rights of ACCESS to data and SOURCE documents

## Data access

The sponsor is responsible for obtaining the agreement of all parties involved in the research to ensure direct access to all places in which the research is performed, to source data, to source documents and to reports for quality control and auditing by the sponsor.
The investigators must make the documents and individual data necessary for monitoring, quality control and auditing of the biomedical research, available to persons who require access to these documents in accordance with legislative and regulatory provisions (Articles R.5121-13 and L.1121-3 of the Code of Public Health).

## Source data

Any original document or object proving the existence or the accuracy of a value or fact recorded during the research is defined as a source document. Investigators should keep TCUS recordings in the medical records. Source data should also be made available for phone interviews.

## Confidentiality of data

In accordance with the existing legislation (Articles L.1121-3 and R.5121-13 of the French code of public health), people with direct access to source data will take all necessary precautions to ensure the confidentiality of information regarding research and participants, in particular with regard to their identity and the results obtained. These people, along with the investigators themselves are subject to professional secrecy.

During the biomedical research or after its termination, the collected participant’s data will be made anonymous before being communicated to the sponsor by the investigating site. In no case should the names or addresses of the subjects appear with medical data transferred to the sponsor. Subjects will be codified with the first letter of their name and surname followed by an incremental number. However, contact details will be transferred to the coordinating center so as to perform centralized phone interviews.

According to study quality control, the sponsor must ensure that each person participating in the research has given written consent for access to their own personal data~~.~~

# QUALITY CONTROL and assurance

## Instructions for data collection

Data will be collected in an electronic case report form. A data dictionary that helps the investigating site will be created for the study. The person/people responsible for filling-in the CRF must be clearly identified in the document describing the distribution of tasks.

## Quality control

A clinical research assistant mandated by the sponsor will regularly visit each investigating center: while the study is being set-up, one or several times during the course of the research depending on the rate of inclusions and at the end of the research. The elements to be reviewed during these visits and the frequency of visits will be defined prior to the start of the study in collaboration with the coordinating team and according to the assessment of the level of risk of the study.

All visits will be the object of a written report. A copy will be forwarded to the Principal Investigator.

## Data management

Data management will be performed by the “Data management and Statistics” unit of Grenoble Alpes University Hospital and follow ongoing procedures. A data management plan will be decided before the beginning of the study.

## Audit and inspection

An audit conducted at the request of the sponsor or an inspection conducted by the health authorities may be carried out at any time by persons independent of those performing the research. It aims to ensure the quality of research, the validity of its results and compliance with the law and regulations.

The auditors / inspectors should have direct access to all source and medical data and any document related to the conduct of the clinical study.
Data confidentiality and anonymity of the subjects will be respected. Investigators must agree to comply with the requirements of the sponsor and the competent authorities regarding an audit or inspection of the research.

An audit may be requested at all stages of the research, from the writing of the protocol to protocol to the publication of results and classification of the data generated or used as part of the research.

# ETHICAL and REGULATORY CONSIDERATIONS

The study will be conducted in accordance with the Declaration of Helsinki (as amended in 2013 in Fortaleza, for full version see http://www.wma.net), the recommendations for Good Clinical Practice (GCP, ICHE6) and any other regulations applicable locally.

The research will be performed in accordance with this protocol. Except in emergency situations requiring the implementation of specific therapeutic procedures, the investigators agree to comply with the protocol in all respects especially regarding obtaining consent and serious adverse event reporting and monitoring.

Grenoble Alps University Hospital, sponsor of this research, has subscribed to an insurance policy for civil liability in accordance with Article L1121-10 of the French Code of Public Health.

The study will not start until the receipt of approval from the Ethics Committee (Comité de Protection des Personnes CPP) and health authorities (National Agency for Safety of Drugs and Health Products, ANSM).

Data recorded during this research are subject to computer processing by the coordinating center and the Data management and Statistics unit of Grenoble Alps University Hospital in accordance with the Law No. 78-17 of 6 January 1978 on computers, files and freedoms amended by Law 2004-801 of August 6, 2004.

This research does not comply with the "Reference Methodology" (MR-001) (Article 54 paragraph 5 of the Act of 6 January 1978 relating to information, files and freedoms). Therefore a specific authorization by the National commission for Informatics and personal liberties (CNIL) will be necessary.This research will be registered on the site <http://clinicaltrials.gov/>

Protocol amendments

Any substantial change, ie. any changes likely to have a significant impact on the protection of persons, the conditions of validity and the results of the research, on the quality and safety of the procedures tested, on the interpretation of the scientific papers that support the conduct of the research or on the conditions in which it is undertaken, will be the subject to a written amendment that is submitted to the sponsor, who must obtain, prior to its implementation, a favorable opinion of the ethics committee (CPP) and authorization by the health authorities (ANSM).

All amendments must be approved by the sponsor, and all the stakeholders in the research who are affected by the change, before submission to the ethics committee (CPP) and the health authorities (ANSM).

All amendments to the Protocol should be made available to all investigators involved in the research. The investigators agree to respect the protocol content. An amendment may also require a new information document and new consent form.

inconvenience/constraints of the study and possible compensation of patients

As a general rule, a patient may not participate simultaneously in a clinical research study which could compromise the TRUST analysis, unless accepted otherwise by the sponsor (case-by-case analysis by the steering committee). There are no supplementary hospital visits, therefore there is no constraint from participating in the study. Subjects will not receive compensation and will not be enrolled in the national register of persons participating in biomedical research.

# Archiving

The following documents concerning this research will be archived in accordance with Good Clinical Practices:

By the investigating physicians:
- **For a period of 15 years following the end of the research**

- The Protocol and any amendments to the Protocol
- The case report forms
- The source documents of participants who signed consent
- All other documents and correspondence relating to the research
- The original copy of the signed informed consent of participants

All these documents are under the responsibility of the investigator for the prescribed archiving period.

By the sponsor:

- **For a period of 15 years following the end of the research**

- The Protocol and any amendments to the Protocol
- All other documents and correspondence relating to the research
- A copy of the signed informed consent of participants
- Documents concerning serious adverse events

All these documents are under the responsibility of the sponsor for the prescribed archiving period.

No transfer or destruction can be made without the agreement of the sponsor. After the regulatory archival period the sponsor will be consulted concerning destruction. All data, all the documents and reports may be subject to audit or inspection.

# PUBLICATIONS

## Scientific communications

Data analysis will carried out by the data management and statistics unit of Grenoble Alps University Hospital in collaboration with the coordinating unit (Research team in Anesthesiology and Critical Care) and the coordinating investigator. This analysis will result in a written report that will be submitted to the sponsor, who shall submit it to the ethics committee (CPP) and the health authorities (ANSM).

Any written or oral communication of research results must receive prior approval of the coordinating investigator and, of any committee established for the research.

The publication of the main results must indicate in the acknowledgements the name of the sponsor, and investigators, methodologists, biostatisticians and data managers who substantially participated in the research, and possibly members of the committees formed for the research, and the name of the funding source. The name of any person contributing to writing or editing the manuscript, if not already in the author list, should also be given in the acknowledgements. Publication will follow international standards of writing and publishing (The Uniform Requirements for Manuscripts of the ICMJE, April 2010) and respect the criteria for authorship.

## Communication of the results to the subjects

According to the law n°2002-303 of March 4, 2002, the participating subjects can be informed, at their request, of the overall results of the research.

## Cessation of the database

Data collection and data management are provided by the associated services. The terms of sale of all or part of the research database are decided by the sponsor who owns the research data and will be subject to a written contract.

1. **DATE ET SIGNATURES**

This protocol was read and approved on the date noted in the header

**Investigators coordinators**

Dr Mélanie SUSTERSIC

Pr Jean-Luc BOSSON

**Investigateurs coordonnateurs**

Anais Kernou

Charlotte Gibert

1. **REFERENCES BIBLIDOCTOR-PATIENT COMMUNICATIONRAPHIQUES**
2. Loi HSPT Article 59 - Loi n°2009-879 du 21 juillet 2009 portant sur la réforme de l’hôpital et relative aux patients, à la santé et aux territoires
3. Arrêté du 26 février 2013 fixant la liste des orientations nationales du développement professionnel continu des professionnels de santé pour l’année 2013
4. Haynes R.B, Taylor D.W et Sackett D.L. Compliance in Health Care. 1979. MD: Johns Hopkins University Press, Baltimore: 1-15
5. Baudrant M. Réflexions sur la place du pharmacien dans l’éducation thérapeutique du patient. Journal de pharmacie clinique. 2008;27(4):201‑4.
6. World Health Organization. (2003). Adherence to long-term therapies, evidence for action. Geneva.
7. Rapport de l’OMS-Europe de 1998 : Therapeutic patient education. Continuing education prdoctor-patient communicationrammes for health care. Providers in the field of prevention of chronic diseases. World Health Organization. 1998, 77 p
8. Haute Autorité de Santé (HAS) et Institut National de Prévention et d'Education pour la Santé (INPES) : "Structuration d'un prdoctor-patient communicationramme d'éducation thérapeutique du patient dans le champ des maladies chroniques", 2007.
9. Article 35 du code de Déontoldoctor-patient communicationie
10. Article R.4127-35 du Code de Santé Publique
11. Wu, E. Q., Guerin, A., Yu, A. P., Bollu, V. K., Guo, A., & Griffin, J. D. (2010). Retrospective real-world comparison of medical visits, costs, and adherence between nilotinib and dasatinib in chronic myeloid leukemia. Curr Med Res Opin, 26 (12), 2861–2869.
12. Morin M. De la recherche à l’intervention sur l’observance thérapeutique : contributions et perspectives des sciences sociales. L’observance aux traitements contre le VIH/Sida : Mesures, déterminants, évolution. Paris, ANRS
13. Ley P. Improving patients’understanding, recall, satisfaction and compliance. In: Broome A, ed.Health psycholdoctor-patient communicationy: processes and applications, 2e ed.London: Chapman, 1995
14. Tarquinio C., Fischer, G.N. & GréGASACire, A. La compliance chez des patients atteints par le VIH : Validation d’une échelle française et mesure de variables psychosociales. Revue Internationale, 2000.
15. Fuertes JN, Mislowack A, Bennett J, Paul L, Gilbert TC, Fontan G, et al. The physician-patient working alliance. Patient Educ Couns. avr 2007;66(1):29‑36.
16. Ciechanowski P, katon W, Russo J et al. The patient-provider relationship: attachment theory an adherence in diabetes. Am J Psychiatry 2001;158:29-35
17. Rainer S. Beck et al. Physician Patient Communication in the Primary Care Office: A Systematic Review. JABFP January–February 2002 Vol. 15 No. 1
18. Kennedy A, Nelson E, Reeves D, Richardson G, Roberts C, Robinson A, et al. A randomised controlled trial to assess the impact of a package comprising a patient-orientated, evidence-based self-help guidebook and patient-centred consultations on disease management and satisfaction in inflammatory bowel disease. Health Technol Assess. 2003;7(28):iii, 1‑113.
19. Sustersic M, Meneau A, Dremont R, Bosson J-L. Fiches d'information patient : quelle méthodoldoctor-patient communicationie ? La Revue du praticien. Médecine générale. 2007 Déc 4;790:1167-68.
20. Sustersic M, Meneau A, Bosson JL; Elaboration de fiches d’information pour les patients en médecine générale. supplément- Rev du Prat 2008; 58.
21. Southall AC, Harris VV. Patient ED turnaround times: a comparative review. Am J Emerg Med. mars 1999;17(2):151‑153.
22. Motifs et trajectoires de recours aux urgences hospitalières, rapport d’une enquête de la DREES en ligne sur www.sfmu.org/documents/ressources/referentiels/er215.pdf‎
23. Thèse de médecine générale : Parcours de soins et motifs de recours aux urgences hospitalières en ligne sur [www.bichat-larib.com/publications.../3327_MEUNIER_Laure_these.pdf](http://www.bichat-larib.com/publications.../3327_MEUNIER_Laure_these.pdf)
24. Morisky DE, Ang A, Krousel-Wood M, Ward HJ. Predictive validity of a medication adherence measure in an outpatient setting. J Clin Hypertens (Greenwich). mai 2008;10(5):348‑354.
25. Ruiz MA, Pardo A, Rejas J, Soto J, Villasante F, Aranguren JL. Development and validation of the « Treatment Satisfaction with Medicines Questionnaire » (SATMED-Q). Value Health. oct 2008;11(5):913‑926.
26. Care Coordination Measures Atlas [Internet]. 2011 [cité 10 sept 2013]. Disponible sur: [http://www.ahrq.GASACv/professionals/systems/long-term-care/resources/coordination/atlas/index.html](http://www.ahrq.gov/professionals/systems/long-term-care/resources/coordination/atlas/index.html)

**ANNEXE 1**

**SCORE d’OBSERVANCE**

**Merci de répondre aux affirmations suivantes :**

Votre médecin vous a-t-il prescrit des médicaments ? OUI 🞎 NON 🞎

Si non, passer directement aux questions II.

1. **Questionnaire d’observance médicamenteuse de Morisky adapté aux patholdoctor-patient communicationies aiguës**
2. Depuis la consultation, avez-vous oublié de prendre vos médicaments ?

OUI 🞎 NON 🞎

1. Depuis la dernière consultation, avez-vous été en panne de médicament (vous n’êtes pas allez les chercher à la pharmacie ou n’en aviez pas assez) ?

OUI 🞎 NON 🞎

1. Vous est-il arrivé de prendre votre traitement avec du retard par rapport à l’heure souhaitée ?

OUI 🞎 NON 🞎

1. Vous est-il arrivé de ne pas prendre votre traitement parce que certains jours votre mémoire vous fait défaut ?

OUI 🞎 NON 🞎

1. Vous est-il arrivé de ne pas prendre votre traitement parce que vous aviez l’impression que votre traitement vous fait plus de mal que de bien ?

OUI 🞎 NON 🞎

1. Pensez-vous que vous avez eu trop de traitements à prendre ?

OUI 🞎 NON 🞎

**---------------------------------------------------------------------------------------------------------------------------**

**B- Questionnaire d’observance globale :**

**I- Observance des prescriptions médicamenteuses**

**Les questions qui suivent concernent les jours qui ont suivi la consultation :**

1. Avez-vous pris l’ensemble du traitement proposé ?

1 (non) 2 (plutôt non) 3 (plutôt oui) 4 (oui, tout à fait)

*Si non ou plutôt non :*

Il vous est arrivé de ne pas prendre l’un ou plusieurs de vos médicaments :

- Parce que vous avez oublié : oui non

- Parce que le traitement est trop complexe: oui non

- A cause d’effets secondaires: oui non

- Parce vos médicaments vous font plus de mal que de bien : oui non

- Parce vous pensiez qu’il n’était pas utile ou adapté: oui non

- Parce que vous sentiez déjà une amélioration : oui non

- Parce que vous n’êtes pas allé chercher les médicaments à la pharmacie : oui non

Si oui : pourquoi : ……………………………………………………………………………….

2. Avez-vous respecté les doses prescrites ?

1 (non) 2 (plutôt non) 3 (plutôt oui) 4 (oui, tout à fait)

3. Avez-vous respecté les modalités de prises (l’heure, à jeun, avant les repas) ?

1 (non) 2 (plutôt non) 3 (plutôt oui) 4 (oui, tout à fait)

4. Avez-vous pris des médicaments autres que ceux prescrits pas votre médecin ?

1 (non) 2 (plutôt non) 3 (plutôt oui) 4 (oui, tout à fait)

Si oui ou plutôt oui :

4bis. Quels autres médicaments avez-vous pris ? …………………………………………..

4ter. Est-ce que ces médicaments ont été prescrits par un médecin ? Oui / Non

***x-1- Question subsidiaire de concordance :***

D’une manière générale, est-ce une décision volontaire de votre part d’avoir pris ou non votre traitement :

1 (non) 2 (plutôt non) 3 (plutôt oui) 4 (oui, tout à fait)

**II- Observance des prescriptions non médicamenteuses (examens complémentaires)**

Votre médecin vous a-t-il prescrit des examens complémentaires et/ou proposé un suivi  et/ou orienté chez un spécialiste ? OUI 🞎 NON 🞎

*Si non*, question non comptabilisée.

*Si oui :* les avez-vous réalisés ?

(Pas du tout) 1 2 3 4 (Complètement)

**III- Observance des consignes/ conseils hygiéno-diététiques et de conduite à tenir selon l’évolution**

1. Votre médecin vous a-t-il donné des conseils à respecter ? OUI 🞎 NON 🞎

(cf.liste ci-dessous pour vous aider)

Par exemple, conseils concernant :

- L’alimentation et/ ou la gestion du poids

- Vos activités habituelles

- L’exercice physique

- Le tabagisme et l’arrêt du tabac

- La consommation d’alcool (ex : diminution si consommation excessive> 2 verres/ jour et % de réussite de l’intention)

- La consommation d’eau (adaptation de la consommation d’eau aux besoins et % de réussite de l’intention).

- Les moyens d’éviter l’aggravation des symptômes, la récidive, la transmission

- Quand reconsulter

*Si non : question non comptabilisée et questions 2, 3, x3 non posées*

*Si oui :*

1. Avez-vous appliqué les conseils donnés et/ou modifié certaines habitudes suite à la consultation*?*

(Pas du tout) 1 2 3 4 (Complètement)

1. Certaines informations données par votre médecin pouvaient-elles intéresser vos proches ?

OUI 🞎 NON 🞎

*Si non,* question non comptabilisée.

*Si oui,* leur avez-vous transmis ces informations ?

(Pas du tout) 1 2 3 4 (Complètement)

x-2- ***Question subsidiaire de concordance :***

D’une manière générale, est-ce une décision volontaire de votre part d’avoir suivi ou non les conseils donnés par votre médecin ?

1 (non) 2 (plutôt non) 3 (plutôt oui) 4 (oui, tout à fait)

**IV- Comportement de consommation de soins**

1. Après la consultation, avez-vous eu besoin d’un nouvel avis médical pour le même problème ?

1 (non) 2 (plutôt non) 3 (plutôt oui) 4 (oui, tout à fait)

*Si oui :*

- Avez-vous consulté à nouveau votre médecin ? OUI 🞎 NON 🞎

- Avez-vous consulté un autre médecin ? OUI 🞎 NON 🞎

- Avez-vous consulté un service d’urgences ? OUI 🞎 NON 🞎

- Avez-vous appelé le centre 15 concernant le même problème ? OUI 🞎 NON 🞎

2. Le médecin vous a-t-il donné des informations sur « quand reconsulter » ? OUI 🞎 NON 🞎

Si oui :

2 bis. Selon vous, le fait d’avoir pris un nouvel avis médical ou non, vous parait-il en accord avec les recommandations de votre médecin ?

(Pas du tout) 1 2 3 4 (complètement)

**** x-3-Question subsidiaire de concordance :***

D’une manière générale, est-ce une décision volontaire de votre part d’avoir suivi ou non les recommandations de votre médecin sur « quand reconsulter » ?

1 (non) 2 (plutôt non) 3 (plutôt oui) 4 (oui, tout à fait)

SYSTEME de COTATION :

Le score d’observance médicamenteuse selon Morisky est coté de 0 à 5

Le score d’observance globale proposé par notre équipe comprend 4 sous-dimensions cotées chacune de 1 à 4 selon une échelle de Likert. Le score étant est la médiane de sous-dimensions. Total de 4 à 16/ 16

- L’observance médicamenteuse ;
- L’observance des prescriptions non médicamenteuses ;
- L’observance des conseils hygiéno-diététiques ;
- L’observance de recours au système de soins ;

**ANNEXE 2**

Questionnaire évaluant la communication médecin malade

1-Le médecin vous a-t-il écouté attentivement pendant la consultation ?

□Non □ Plutôt non □ Plutôt oui □ Oui

2-Le médecin vous a-t-il interrompu pendant que vous parliez ?

□ Non □ Plutôt non □ Plutôt oui □ Oui

3- Le médecin vous a-t-il encouragé à vous exprimer ? □Non □ Plutôt non □ Plutôt oui □ Oui

4- Le médecin vous a-t-il bien examiné ?

□ Non □ Plutôt non □ Plutôt oui □ Oui

5- Vous-êtes-vous senti compris par le médecin ?

□ Non □ Plutôt non □ Plutôt oui □ Oui

6- Est-ce que le médecin s’est exprimé de façon compréhensible ?

□ Non □ Plutôt non □ Plutôt oui □ Oui

7- Avez-vous eu l’impression d’avoir eu toutes les informations nécessaires ?

□ Non □ Plutôt non □ Plutôt oui □ Oui

8- Le médecin vous a-t-il expliqué les avantages et inconvénients du traitement ?

□ Non □ Plutôt non □ Plutôt oui □ Oui

9- Le médecin vous a-t-il impliqué dans la prise de décision ?

□ Non □ Plutôt non □ Plutôt oui □ Oui

10- D’après vous le médecin a-t-il eu une attitude et un discours rassurants ?

□ Non □ Plutôt non □ Plutôt oui □ Oui

11- D'après vous, le médecin a-t-il été globalement respectueux ?

□ Non □ Plutôt non □ Plutôt oui □ Oui

- Respect de votre intimité ? □ oui □ non
- Respect de vos croyances ? □ oui □ non
- Garde confidentielles les informations données ? □ oui □ non
- Ne porte pas de jugement ? □ oui □ non

- Respect de votre corps lors de l’examen clinique □ oui □ non

12- Avez-vous confiance en ce médecin ?

□ Non □ Plutôt non □ Plutôt oui □ Oui

13- D'après vous, le médecin vous a-t ‘il dit toute la vérité ?

□ Non □ Plutôt non □ Plutôt oui □ Oui

14- Le médecin s’est-il assuré que vous aviez bien compris ses explications?

□ Non □ Plutôt non □ Plutôt oui □ Oui

15- Le médecin a-t ‘il répondu à toutes vos attentes et/ou préoccupations?

□ Non □ Plutôt non □ Plutôt oui □ Oui

**SCORE TOTAL : / 60**

Chacun des items est coté selon la réponse 1, 2, 3 ou 4.

Score côté sur 60 avec un score minimum à 15/60 et un maximum à 60/60

**ANNEXE 3**

Méthodoldoctor-patient communicationie d’élaboration du questionnaire de communication médecin-malade

**Objectif :** **Élaboration d’une échelle et de son score évaluant la qualité de la communication médecin malade à partir d’une revue de la littérature**

Travail universitaire effectué avec l’appui méthodoldoctor-patient communicationique du CIC (Pr JLBosson), du CNRS (équipe ThEMAS) et de la faculté de psycholdoctor-patient communicationie (Dr Aurélie Gauchet, psycholdoctor-patient communicationie de la Santé).

**Méthodoldoctor-patient communicationie :**

La première étape a consisté en une recherche biblidoctor-patient communicationraphique sur les bases de données « pubmed » et « psychinfo » ainsi que les sites institutionnels français (HAS), anglais (NHS) et américain (AHRQ).

Les mots clés étaient **«**physician-patient relations », « psychometrics », « questionnaires », « scale », « communication ».

Pour élaborer une échelle multidimensionnelle évaluant la qualité de la communication médecin malade, nous nous sommes appuyés sur le modèle de communication suivant: dans un contexte donné commun, émission d’une information par le médecin avec des mots et un discours intelligible -> réception par le patient qui confirme au médecin que le message a bien été reçu. Ce qui peut être résumé de la manière suivante : émission -> information -> réception-> feedback.

Les échelles ont été sélectionnées selon les critères d’inclusion suivants : échelles validées ; en langue Française ou Anglaise ; évaluant la communication médecin malade (échelles de communication médecin malade, de relation médecin malade, d’écoute, de confiance, d’empathie).

Dans notre choix de modèle théorique, les conséquences de la communication sur le comportement (échelles d’observance), le psychisme (échelles de satisfaction, anxiété, dépression, auto efficacité personnelle etc.) ou les symptômes du patient (échelles de douleur…etc.) ont été considérées comme étant extrinsèques à la communication médecin malade. Les échelles mesurant ces dimensions ont donc été exclues. Les autres critères d’exclusion étaient : les échelles spécifiques des patholdoctor-patient communicationies chroniques ; celles mesurant des dimensions hors champ de la consultation (organisation du système de soins, temps d’attente dans la salle d’attente…etc.). Au total, vingt échelles ont été retenues.

La seconde étape a consisté à extraire à partir des échelles retenues tous les items pertinents sur le sujet sans préjuger des dimensions auxquelles ils appartenaient. Ensuite, lorsque deux ou plusieurs items étaient redondants, un seul a été retenu, ce qui nous a permis d’aboutir à un questionnaire à la fois exhaustif et synthétique. Cette démarche (inverse par rapport à la démarche communément utilisée qui aurait été de choisir un modèle théorique en psycholdoctor-patient communicationie, de définir ensuite les dimensions caractérisant le modèle et enfin de rédiger les items caractérisant chacune des dimensions) a permis d’éviter plusieurs écueils.

Tout d’abord celui de créer une énième échelle parmi tant d’autres sans prendre en compte les travaux antérieurs réalisés sur le sujet. Ensuite celui d’avoir des items chevauchant potentiellement plusieurs dimensions.

En effet, les dimensions ayant des définitions variables selon les équipes de chercheurs, le choix d’un modèle théorique implique une part d’arbitraire. Aucun modèle théorique n’étant parfait, il est forcément réducteur de choisir un modèle plutôt qu’un autre.

Notre classification par dimensions a eu pour seul objectif de faciliter le tri des items et aucunement d’attribuer à chaque item une dimension. Notre échelle et/ou questionnaire fonctionne donc indépendamment des dimensions qui le composent.

La troisième étape a consisté à reformuler les items afin que les réponses aux questions puissent être mesurées sur une échelle de Likert allant de 1 à 4.

Une étude qualitative réalisée sur une vingtaine de patients est actuellement en cours.

Elle consiste à demander au patient pour chacun des items :

- une reformulation de l’item pour s’assurer qu’il l’a bien compris;
- savoir si l’item lui paraît pertinent par une question ouverte;
- savoir si l’item englobe bien tous les aspects qu’il était censé englobé lorsqu’il a été sélectionné, afin de ne pas omettre de notion.

Elle a pour but de vérifier la bonne compréhension du questionnaire par les patients avant d'aborder la seconde étape, quantitative, qui se fera sur environ 200 patients, afin de valider l'échelle et ses propriétés psychométriques.

**Références biblidoctor-patient communicationraphiques**

- Atlas AHRQ : <http://www.childhealthdata.org/docs/drc/ahrq-care-coordination-atlas-dec-2010.pdf>
- Mack JW et al. [Measuring therapeutic alliance between oncoldoctor-patient communicationists and patients with advanced cancer: the Human Connexion Scale.](http://www.ncbi.nlm.nih.gov/pubmed/19484795) Cancer. 2009 Jul
- Jane Doctor-patient communicationden. Psycholdoctor-patient communicationie de la santé. Edition De Boeck 2004

Claude Richard et Marie-Thèrèse Lussier. La communication professionnelle en santé. Édition Erpi. 2002

- Rainer S. Beck et al. Physician Patient Communication in the Primary Care Office: A Systematic Review. JABFP January–February 2002 Vol. 15 No. 1

**ANNEXE 4**

**Questionnaire général**

Code d’identification du patient :

Date de consultation aux urgences :

Date(s) d’appel téléphonique :

Nous souhaitons recueillir votre opinion sur votre dernière consultation aux urgences. Cet entretien va durer environ 15 minutes.

Nous vous remercions du temps que vous allez prendre pour répondre à cette enquête.

Vos réponses sont anonymes.

**A/ Données sociodémdoctor-patient communicationraphiques**

Tout d’abord, quelques questions pour mieux vous connaître :

1. Quel âge avez-vous ?
2. Quel est votre niveau d’études ?

□ Collège □ Bac □ Bac +2 □ Études supérieures après Bac +2 □ autres

1. Travaillez-vous dans le milieu médical ?

□ Oui □ Non

1. A quelle catéGASACrie socio-professionnelle appartenez-vous ?
2. Quelle est votre situation familiale : célibataire / en couple / marié ;

et nombre d’enfants :

**B/ Score de Satisfaction :**

Suite à votre consultation dans le service d’urgences :

- Quels sont les éléments dont vous êtes satisfaits ?

……………………………………………………………………………………………………………………………………………………………………………………………….

- Quels sont les éléments qui vous ont déplu ?

……………………………………………………………………………………………………………………………………………………………………………………………….

1. Diriez-vous que vous êtes satisfait des conditions d’accueil, hors paramètres médicaux (accueil, nourriture, délais d’attente) dont vous avez bénéficié ?

□ Non □ Plutôt non □ Plutôt oui □ Oui

1. Diriez-vous que vous êtes satisfait des soins reçus et de la prise en charge par l’équipe paramédicale (infirmier(e)s, aides-soignants, ASH) ?

□ Non □ Plutôt non □ Plutôt oui □ Oui

1. Diriez –vous que vous êtes satisfait de la prise en charge par le médecin que vous avez rencontré ?

□ Non □ Plutôt non □ Plutôt oui □ Oui

1. De façon globale, diriez-vous que vous êtes satisfait de votre consultation aux urgences ?

□ Non □ Plutôt non □ Plutôt oui □ Oui

1. Recommanderiez-vous ce service à votre famille et vos amis ?

□ Non □ Plutôt non □ Plutôt oui □ Oui

Chacun des items est coté selon la réponse : 1(non), 2, 3 ou 4 (oui).

Score coté sur 20 avec un minimum de 5/20 et un maximum de 20/20.

**ANNEXE 5**

**Cher confrère,**

Nous vous remercions de participer à cette étude, qui a plusieurs objectifs :

1/ Première étape (Hiver 2013) :

- Faire un **état des lieux des niveaux de satisfaction des patients, de communication médecin-malade, et d’observance thérapeutique** dans un service d’urgences;
- Valider deux scores (communication médecin-malade et observance thérapeutique) génériques, dans le but ensuite de pouvoir les utiliser comme outil d’évaluation de la qualité de toute consultation.

2/ Deuxième étape (Courant 2014) visant à améliorer la prise en charge des patients, à l’aide d’un outil : les **Fiches Information Patients** (FIP).

- Évaluer la faisabilité de l’usage de ces FIP dans un service d’urgences ;
- Évaluer leur impact sur la communication médecin-malade et l’observance thérapeutique.

Cette étude s’inscrit ainsi dans une démarche **d’Evaluation des Pratiques Professionnelles et de Développement Professionnel Continu**, qui est rendu obligatoire par la loi HPST du 21 Juillet 2009.

Durant cette période, nous vous remercions de bien vouloir nous aider en suivant la démarche ci-dessous :

**Patient répondant aux critères d’inclusion :**

Patient majeur ou responsable légal d’un mineur ≥ 15 ans et 3mois, lettré, joignable par téléphone dans les dix jours qui suivent,

Consultant pour : Pyélonéphrite Aiguë ou Entorse de Cheville

Consultation Habituelle

**Explication brève de l’étude au patinet + Remise du « Formulaire d’information et non opposition » et de la « Fiche d’inclusion »**

**Accord** du patient pour **Refus** du patient de participer

être contacté par téléphone et participer à à l’étude l’étude

**INCLUSION : NON INCLUSION :**

Remplir la fiche d’inclusion Remplir la fiche d’inclusion **Avec les données** nominatives du patient **Sans les données** nominatives du patient

En cochant la patholdoctor-patient communicationie concernée En cochant « refus de contact téléphonique »

**Mettre la fiche d’inclusion dans la corbeille prévue à cet effet**

Vous recevrez bien sûr les résultats de cette étude.

Si vous avez la moindre interrdoctor-patient communicationation, vous pouvez nous joindre au 06 64 36 42 77 (Laure),

ou au 06 72 31 23 94 (Amélie).

Merci encore pour votre participation à cette étude

Laure GASACNNET et Amélie DUVERT (internes en Médecine Générale)

**ANNEXE 6**

## FORMULAIRE D’INFORMATION ET DE NON-OPPOSITION

***Document constitué en application du Code de Santé Publique.***

Madame, Monsieur,

Vous consultez les urgences ce jour pour une entorse de cheville ou une pyélonéphrite aiguë [barrer la mention non applicable].

Nous vous proposons de participer à une recherche médicale dont le but est d’évaluer la qualité des consultations aux urgences, notamment en termes de communication médecin-malade, et de satisfaction des patients. Les résultats de cette recherche pourraient nous aider, par des fiches d’information destinés aux patients, à améliorer ces points dans le futur.

**Déroulement de l’étude :**

Le médecin que vous avez rencontré lors de votre consultation ce jour, vous a prescrit un traitement et/ou des conseils à suivre.

Vous serez ensuite contacté(e) par téléphone dans une dizaine de jours par un des médecins coordonnateurs de l’étude. Cela durera une dizaine de minutes. Cet appel téléphonique garantit le secret médical. En cas de répondant intermédiaire, aucune information médicale ne sera donnée.

Votre participation ne modifie en rien les pratiques médicales vous concernant (examens, traitements).

Afin de pouvoir vous joindre, nous avons besoin de vos coordonnées. Nous vous demandons également les coordonnées d’une personne de confiance, si vous en avez désigné une, pour permettre aux coordonnateurs d’effectuer l’enquête si vous-même n’êtes pas en mesure de répondre.

**Confidentialités des données vous concernant :**

Dans le cadre de cette étude les règles usuelles de confidentialité seront respectées.

Les données médicales, vous concernant, seront centralisées dans un fichier informatique à des fins de traitement statistique. Elles resteront strictement confidentielles. Ces données seront transmises au Centre d’Investigation Clinique du CHU de Grenoble ou aux personnes agissant pour son compte.

Suite à l’enquête téléphonique, vos coordonnées personnelles seront détruites. Vos réponses resteront anonymes. Elles se verront attribuer un code comportant un numéro et les initiales de votre nom et prénom, qui lui seul sera informatisé.

Conformément aux dispositions de la loi relative à l’informatique aux fichiers et aux libertés, vous disposez d’un droit d’accès et de rectification. Vous disposez également d’un droit d’opposition à la transmission de vos données couvertes par le secret professionnel susceptibles d’être utilisées et traitées dans le cadre de cette recherche, en contactant les médecins coordonnateurs de l’étude nommés ci-dessous.

Vous avez bien sûr le droit de refuser de participer à cette recherche, il vous suffit de remplir le bas de ce document et de le remettre au médecin qui vous prend en charge, ce refus ne changera en rien vos relations avec les médecins. Vous avez en outre la possibilité de vous y opposer à tout moment pour la raison de votre choix, sans encourir aucune responsabilité, sans atteinte à la qualité des soins que vous recevrez ultérieurement.

En coopérant à cette enquête, vous participerez à la recherche médicale. Vous aidez les médecins français à utiliser de nouveaux moyens d’information et nous vous en remercions.

Je soussigné(e) Mme, Mr….………………………

Ne souhaite pas participer à l’étude « Etat des lieux dans un service d’Urgence dans un but d’Amélioration des Pratiques Professionnelles ».

Aucune donnée concernant mon dossier médical ne sera prise en compte pour l’étude. J’ai bien compris que le fait de ne pas participer à l’étude ne changera en rien la façon dont je serai prise en charge.

Fait le …………………………………, à …………………………………,

Signature :

*Nous vous remercions pour votre participation à cette étude*

**
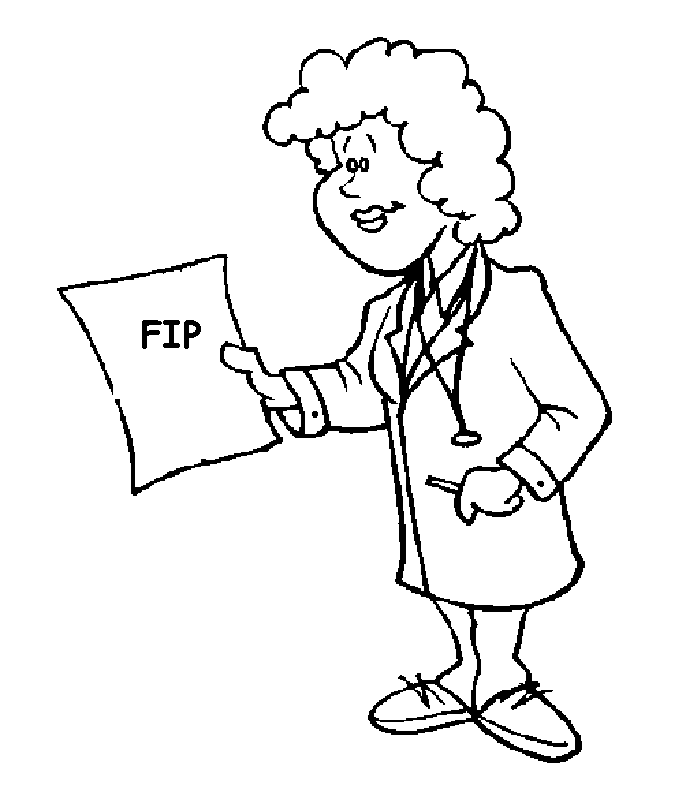
**

**DUVERT Amélie et GASACNNET Laure**

**Internes en Médecine Générale**

**Coordonnateurs de l’étude**

## ANNEXE 6 bis

## FORMULAIRE D’INFORMATION ET DE NON-OPPOSITION

Pour responsable légal d’un mineur

***Document constitué en application du Code de Santé Publique.***

Madame, Monsieur,

Votre enfant, (ou le mineur dont vous êtes le responsable légal) a consulté les urgences ce jour pour une entorse de cheville ou une pyélonéphrite aiguë [barrer la mention non applicable].

Nous vous proposons de participer à une recherche médicale dont le but est d’évaluer la qualité des consultations aux urgences, notamment en termes de communication médecin-malade, et de satisfaction des patients. Les résultats de cette recherche pourraient nous aider, par des fiches d’information destinés aux patients, à améliorer ces points dans le futur.

**Déroulement de l’étude :**

Le médecin que vous avez rencontré lors de votre consultation ce jour, a prescrit un traitement et/ou des conseils à suivre à votre enfant.

Vous serez ensuite contacté(e) par téléphone dans une dizaine de jours par un des médecins coordonnateurs de l’étude. Cela durera une dizaine de minutes. Cet appel téléphonique garantit le secret médical. En cas de répondant intermédiaire, aucune information médicale ne sera donnée.

Votre participation ne modifie en rien les pratiques médicales concernant votre enfant (examens, traitements).

Afin de pouvoir vous joindre, nous avons besoin de vos coordonnées. Nous vous demandons également les coordonnées d’une personne de confiance, si vous en avez désigné une, pour permettre aux coordonnateurs d’effectuer l’enquête si vous-même n’êtes pas en mesure de répondre.

**Confidentialités des données concernant votre enfant :**

Dans le cadre de cette étude les règles usuelles de confidentialité seront respectées.

Les données médicales concernant votre enfant, seront centralisées dans un fichier informatique à des fins de traitement statistique. Elles resteront strictement confidentielles. Ces données seront transmises au Centre d’Investigation Clinique du CHU de Grenoble ou aux personnes agissant pour son compte.

Suite à l’enquête téléphonique, vos coordonnées personnelles seront détruites. Vos réponses resteront anonymes. Elles se verront attribuer un code comportant un numéro et les initiales de votre nom et prénom, qui lui seul sera informatisé.

Conformément aux dispositions de la loi relative à l’informatique aux fichiers et aux libertés, vous disposez d’un droit d’accès et de rectification. Vous disposez également d’un droit d’opposition à la transmission de vos données couvertes par le secret professionnel susceptibles d’être utilisées et traitées dans le cadre de cette recherche, en contactant les médecins coordonnateurs de l’étude nommés ci-dessous.

Vous avez bien sûr le droit de refuser de participer à cette recherche, il vous suffit de remplir le bas de ce document et de le remettre au médecin qui vous prend en charge, ce refus ne changera en rien vos relations avec les médecins. Vous avez en outre la possibilité de vous y opposer à tout moment pour la raison de votre choix, sans encourir aucune responsabilité, sans atteinte à la qualité des soins que vous recevrez ultérieurement.

En coopérant à cette enquête, vous participerez à la recherche médicale. Vous aidez les médecins français à utiliser de nouveaux moyens d’information et nous vous en remercions.

Je soussigné(e) Mme, Mr….………………………, responsable légal de l’enfant ………………………………………….. ……………………………………………………………..

ne souhaite pas participer à l’étude « Etat des lieux de la communication et de la satisfaction dans un service d’Urgence dans un but d’Amélioration des Pratiques Professionnelles ».

Aucune donnée concernant le dossier médical de mon enfant ne sera prise en compte pour l’étude. J’ai bien compris que le fait de ne pas participer à l’étude ne changera en rien la façon dont mon enfant sera pris en charge.

Fait le …………………………………, à …………………………………,

Signature :

*Nous vous remercions pour votre participation à cette étude*

**
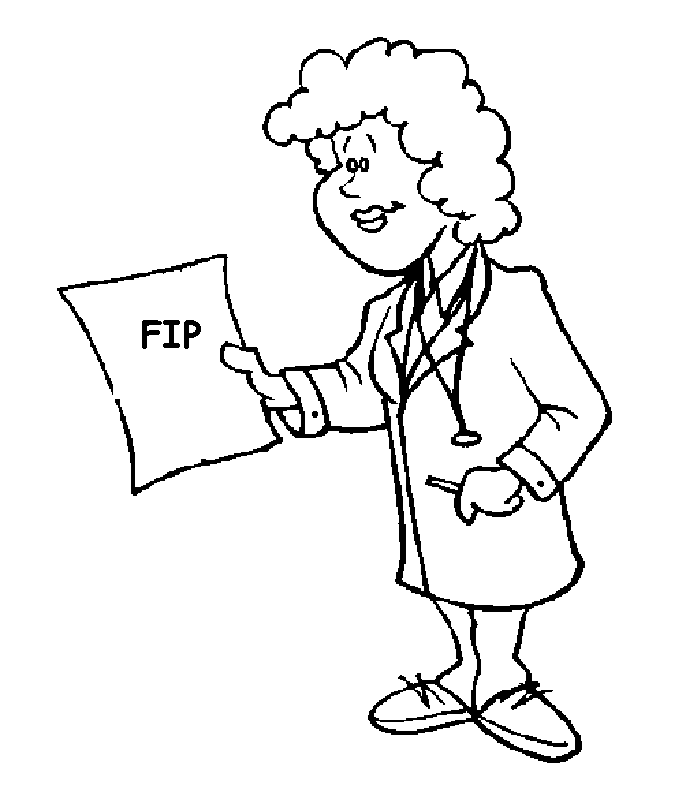
**

**DUVERT Amélie et GASACNNET Laure**

**Internes en Médecine Générale**

**Coordonnateurs de l’étude**

**ANNEXE 7**

**FICHE d’INCLUSION**

**(à remettre dans la corbeille prévue à cet effet)**

**Date de la consultation d’inclusion :**

**Refus de contact téléphonique :**

(si case cochée, ne pas remplir les données nominatives)

**Données nominatives du patient (non informatisées) ou du responsable légal si mineur**

Nom :

Prénom :

Sexe :

Téléphone fixe patient: / / / / / / / / / / /

Téléphone portable patient : / / / / / / / / / / /

Téléphone d’un proche (désigné comme personne de confiance) : / / / / / / / / / / / (précisez : voisin, parent…)

**Jour de la semaine et créneaux horaires préférés par le patient pour être contacté :**

**Code d’identification du patient :**

2 premières lettres du nom du patient : / / /

Première lettre du prénom du patient : / /

**Patholdoctor-patient communicationie concernée (entourer la mention utile)**

- Entorse de cheville
- Pyélonéphrite aigue
